# Interlayer Coordination of Pd–Pd Units in Exfoliated Black Phosphorus

**DOI:** 10.1021/jacs.1c01754

**Published:** 2021-06-29

**Authors:** Matteo Vanni, Marco Bellini, Silvia Borsacchi, Lucia Calucci, Maria Caporali, Stefano Caporali, Francesco d’Acapito, Marco Geppi, Andrea Giaccherini, Andrea Ienco, Gabriele Manca, Antonio Massimiliano Mio, Giuseppe Nicotra, Werner Oberhauser, Manuel Serrano-Ruiz, Martina Banchelli, Francesco Vizza, Maurizio Peruzzini

**Affiliations:** † Institute for the Chemistry of Organometallic Compounds (CNR-ICCOM), Via Madonna del Piano 10, 50019 Sesto Fiorentino, Italy; ‡ Department of Biotechnology, Chemistry and Pharmacy, University of Siena, 53100 Siena, Italy; § Institute for the Chemistry of Organometallic Compounds (CNR-ICCOM), SS Pisa, Via Moruzzi 1, 56124 Pisa, Italy; ∥ Center for Instrument Sharing of the University of Pisa (CISUP), Lungarno Pacinotti 43/44, 56126 Pisa, Italy; ⊥ Department of Industrial Engineering, University of Florence, Via di S. Marta 3, 50139 Firenze, Italy; # CNR-IOM-OGG c/o European Synchrotron Radiation Facility, 71 Avenue des Martyrs, CS 40220, 38043 Grenoble Cedex 9, France; ∇ Department of Chemistry and Industrial Chemistry (DCCI), University of Pisa, Via Moruzzi 13, 56121 Pisa, Italy; ● Department of Earth Sciences, University of Florence, Via La Pira 4, 50121 Firenze, Italy; ○ Institute for Microelectronics and Microsystems (CNR-IMM), VIII strada 5, I-95121 Catania, Italy; △ Institute of Applied Physics “Nello Carrara” (CNR-IFAC), Via Madonna del Piano 10, 50019 Sesto Fiorentino, Italy

## Abstract

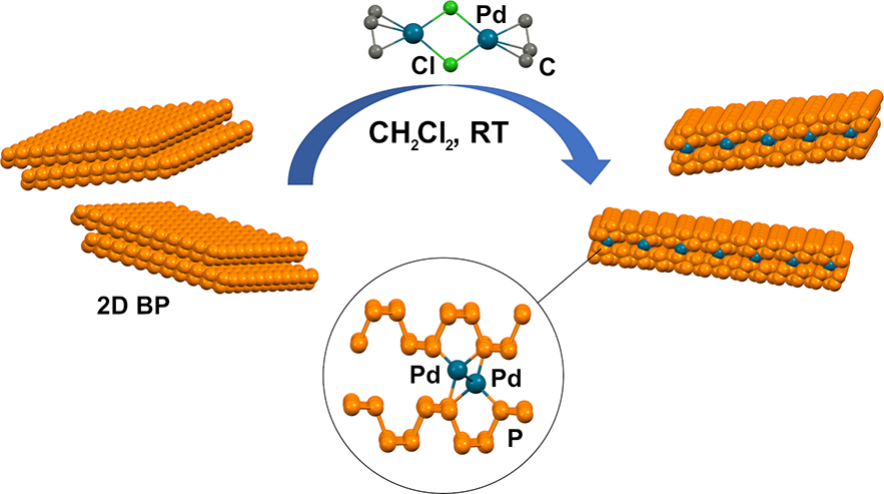

The chemical functionalization of 2D exfoliated black phosphorus (2D BP) continues to attract great interest, although a satisfactory structural characterization of the functionalized material has seldom been achieved. Herein, we provide the first complete structural characterization of 2D BP functionalized with rare discrete Pd_2_ units, obtained through a mild decomposition of the organometallic dimeric precursor [Pd(η^3^-C_3_H_5_)Cl]_2_. A multitechnique approach, including HAADF-STEM, solid-state NMR, XPS, and XAS, was used to study in detail the morphology of the palladated nanosheets (Pd_2_/BP) and to unravel the coordination of Pd_2_ units to phosphorus atoms of 2D BP. In particular, XAS, backed up by DFT modeling, revealed the existence of unprecedented interlayer Pd–Pd units, sandwiched between stacked BP layers. The preliminary application of Pd_2_/BP as a catalyst for the hydrogen evolution reaction (HER) in acidic medium highlighted an activity increase due to the presence of Pd_2_ units.

## 1 Introduction

The field of 2D materials has been continuously flourishing over the last 10 years, leading to the discovery of many graphene-related compounds, including MXenes,^1^ 2D transition metal dichalcogenides,^2^ group 14 2D allotropes (silicene, germanene, stannene),^3^ and layered pnictogens (phosphorene, arsenene, antimonene, bismuthene).^4,5^ Black phosphorus (BP) in particular has experienced a true renaissance since 2014, when its exfoliation was simultaneously reported by the groups of Zhang and Ye.^6,7^ The remarkable properties of BP include a layer-dependent direct band gap (going from 0.35 eV in the bulk to 2.2 eV in the monolayer “phosphorene”), an ultrahigh carrier mobility (1000 cm^2^ V^–1^ s^–1^ at room temperature), and a thermoelectric behavior.^8−10^ Several promising applications have emerged in distinct fields, including microelectronics,^11,12^ sensor technology,^13−15^ energy conversion,^16,17^ catalysis,^18−20^ and nanomedicine.^21−25^

Unlike graphite, its carbon congener, the sp^3^ hybridization of BP phosphorus atoms gives rise to a puckered layer conformation, in which every P atom bears a lone pair, suggesting a feasible functionalization of exfoliated BP (2D BP). To date, great efforts have been made to modify the surface of BP. Though surface decoration with metal nanoparticles has been extensively developed,^18,26−28^ only a few studies have addressed its reactivity with molecular fragments, mainly organic molecules.^29^ Some established protocols include edge functionalization with C_60_ buckyballs,^30^ reductive activation with alkali metals followed by alkylation with iodides,^31^ surface functionalization with nitrenes,^32,33^ and arylation with diazonium salts,^34,35^ though the last has recently been questioned.^36^ Even scarcer are functionalizations with metal complexes, the main examples concerning the use of TiX_4_^37^ and LnX_3_^38^ salts (Ln = lanthanide, X = sulfonate) as surface modifiers. However, in those reports the structure and bonding properties of the adduct between 2D BP and the metal (M) were scarcely addressed, providing limited evidence of direct P–M bonding and lacking deeper structural investigations. More recently, some of us reported a detailed computational study addressing both the steric and electronic factors ruling the covalent functionalization of 2D BP with different transition metal fragments and Lewis acids^39^ as well as the reactivty with suitable chalcogen transfer reagents.^40^ Currently, major advances in solid state characterization techniques (X-ray absorption spectroscopy, XAS) and local investigation (transmission electron microscopy, TEM, and high-angle annular dark field–scanning TEM, HAADF-STEM), together with *ab initio* modeling, allow an in-depth structural knowledge of low-nuclearity systems, such as single- and double-atom catalysts,^41−45^ rivaling the role of single-crystal X-ray diffraction in molecular science. However, this level of accuracy is seldom encountered within the field of 2D materials, particularly with functionalized BP.

Herein, we investigated the functionalization of 2D BP obtained through its reaction with the organometallic precursor [Pd(η^3^-C_3_H_5_)Cl]_2_ (**1**). The latter is a well-known air-stable dimeric organopalladium complex that easily undergoes opening of the chloride bridge even in the presence of weak σ-donor ligands, while strong donor abilities are mandatory for the stabilization of the allylic moieties.^46^ Thus, given the scarce Lewis basicity of phosphorus atoms in 2D BP,^39^ we speculated that **1** may pave the way to the generation of isolated Pd(0) species (monoatomic or polyatomic) located on the BP layers, upon the decomposition of initially grafted {Pd(C_3_H_5_)Cl} units. A thorough structural characterization of the functionalized material (named Pd_2_/BP), backed up by a sound DFT analysis, revealed the correctness of this hypothesis and corroborated the existence of unprecedented interlayer Pd–Pd diatomic units bridging two “phosphorene” layers.

## 2 Results and Discussion

### 2.1 Structural Characterization

**Figure 1:**
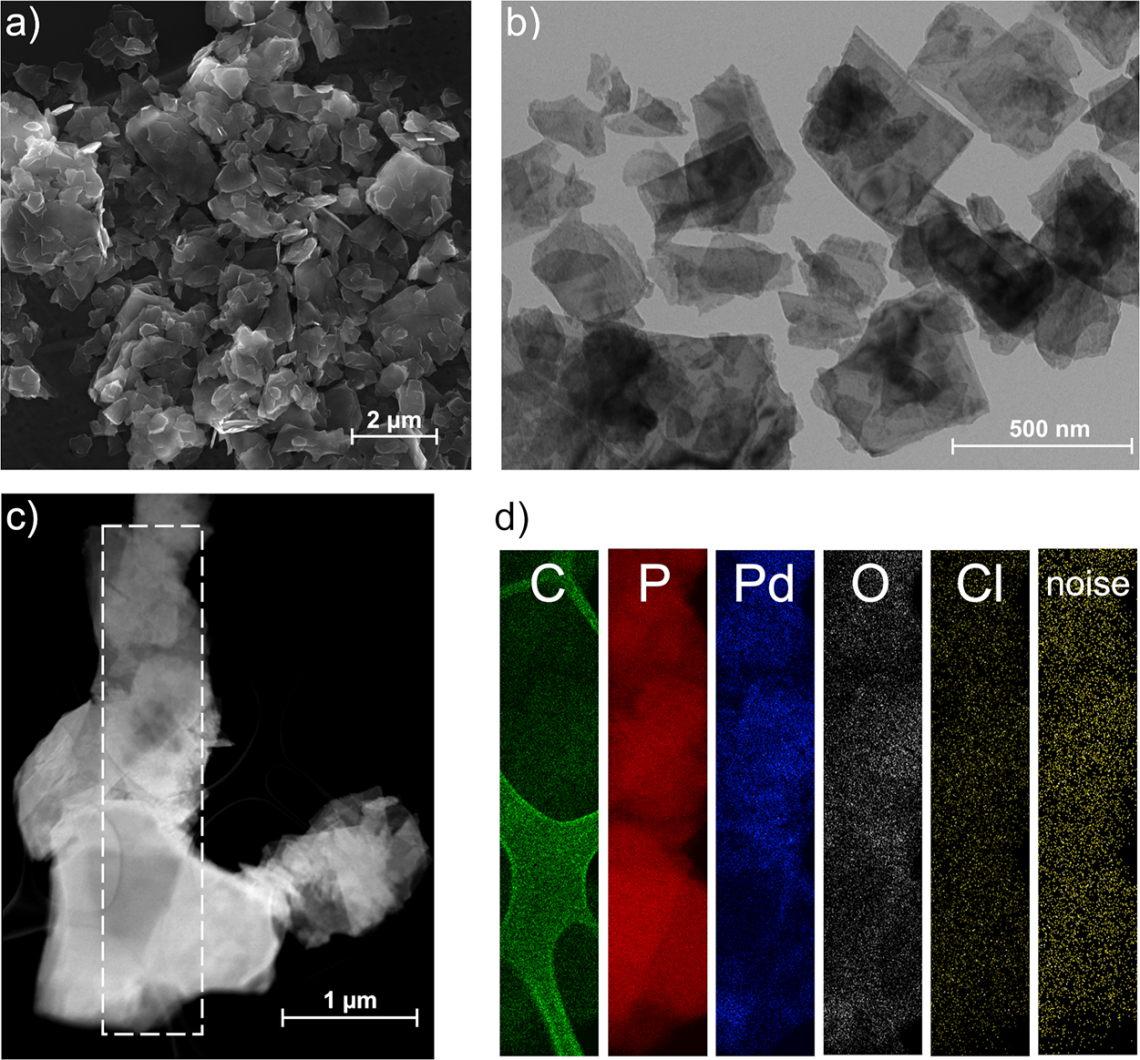
(a) SEM and (b) TEM images of Pd_2_/BP 3%. (c) HAADF-STEM image of a flake aggregate drop-casted on a carbon grid. (d) EDS mapping of the region highlighted in (c). The underlying carbon grid is visible in the C elemental mapping.

The functionalization of 2D BP was carried out under mild reaction conditions, working in dry dichloromethane (DCM) as solvent. The latter, chosen for its innocent and negligible nucleophilic behavior, provides stable dispersions of 2D BP and easily dissolves **1**. Remarkably, immobilization of Pd onto 2D BP occurs easily by stirring a dispersion of the pristine material in DCM together with **1** for 17 h (see the Supporting Information for details). An inductively coupled plasma–atomic emission spectroscopy (ICP-AES) analysis of the isolated material revealed a Pd content of 3.3% (Pd/P mole ratio) when the reaction was carried out at RT and 6.1% when it was performed under reflux. The two samples were named Pd_2_/BP 3% and 6%, respectively. Actually, the amount of Pd immobilized in 2D BP seems inconsistent with a molecular *surface* functionalization, as is easily explained. Our pristine exfoliated material (see the Supporting Information) features flake thicknesses within the range 2–30 nm (corresponding to ca. 5–58 layers). For an ideal exfoliated material consisting of thin BP flakes with a thickness of 10 nm (ca. 19 layers), the ratio between palladium and surface phosphorus atoms (i.e. exposed external atoms, P_surf_) for an experimental metal loading of 3.3% would be Pd/P_surf_ = 0.66: namely, two Pd atoms for every three P_surf_ atoms. These values seem too high for a surface functionalization, unless some Pd aggregate is also present (i.e. Pd nanoparticles (NPs) or PdP_*x*_ phases). Thus, to get insights into the morphology of Pd_2_/BP, electron microscopy studies were carried out on the material. Figure 1a–c shows Scanning electron microscopy (SEM), TEM, and STEM images of Pd_2_/BP, respectively. The BP flakes look perfectly intact after functionalization, keeping their overall morphology unaltered. Notably, no presence of Pd NPs could be detected via TEM.

**Figure 2:**
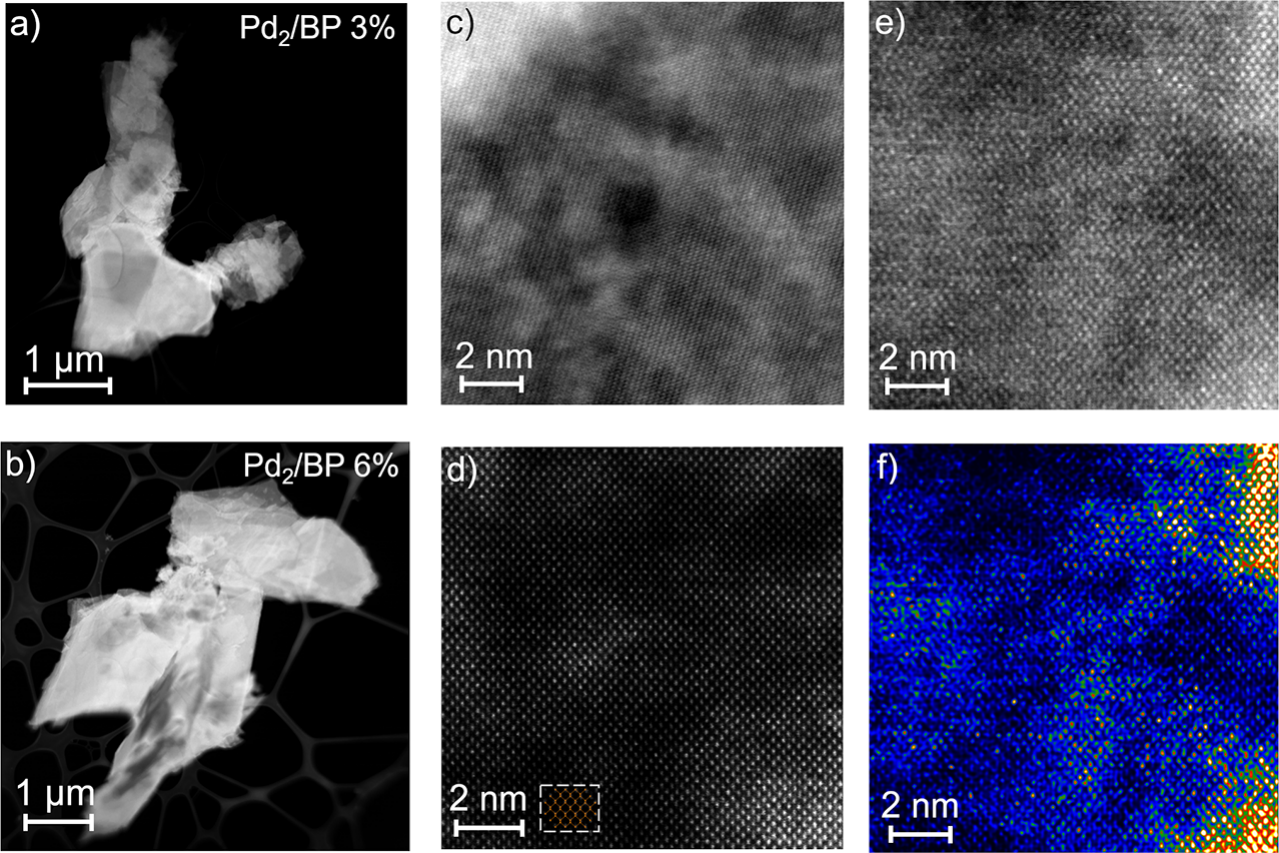
HAADF-STEM characterization of Pd_2_/BP at different Pd loadings. Flakes stacking from (a) Pd_2_/BP 3% and (b) Pd_2_/BP 6% obtained by drop-casting DCM dispersions on a carbon grid. High resolution micrographs of flakes taken from (c) Pd_2_/BP 3% and (d) Pd_2_/BP 6% (FFT filtered). The inset next to the scale bar in (d) shows the schematic atomic arrangement of the BP lattice. Pd-rich areas are distinguished by the higher *Z* contrast (brighter areas). (e) Micrograph taken from a Pd_2_/BP 6% flake (raw data) and (f) corresponding image displayed in false colors (warmer color = higher *Z*).

Energy-dispersive X-ray spectroscopy (EDS) was used to study the elemental composition of Pd_2_/BP on a nanometer scale; the resulting EDS mappings are shown in Figure 1d. As it turned out, Pd is homogeneously distributed within a flake, pointing to a very dispersed form of the metal, possibly on the atomic or polyatomic level. No presence of chlorine was detected in the material, as its integrated EDS signal was below the noise level, ruling out the presence of {Pd(C_3_H_5_)Cl} fragments grafted on the 2D BP surface. Since exceedingly small metal NPs and clusters could be missed under survey TEM analysis, high-resolution morphological and structural investigations were performed via annular dark field microscopy (HAADF-STEM). Figure 2 shows high-resolution micrographs of Pd_2_/BP. The image in Figure 2d was FFT (fast Fourier transform) filtered to reduce the noise, whereas the image in Figure 2f was generated from the raw data of Figure 2e upon FFT filtering and false-color display (warmer colors correspond to higher *Z*). As can be observed, high-*Z* domains (brighter areas) look dispersed in the region under study. Remarkably, the lattice structure of BP is perfectly distinguishable even within high-*Z* regions (i.e. with a higher local concentration of palladium). This can be nicely appreciated from Figure 2d. This finding would be consistent with atomic or molecular functionalization of the flakes, ruling out the presence of both Pd–Pd crystalline domains and Pd-containing amorphous structures such as PdP_*x*_ phosphide species. The latter would otherwise appear superimposed on the lighter BP lattice in the image, making it look distorted or obscured. At the same time, this evidence proves the integrity of the BP lattice upon functionalization.

Electron energy loss spectroscopy (EELS) is a powerful technique for elemental microanalysis, particularly to detect light elements. To further confirm the absence of chlorine in Pd_2_/BP, comparative EELS measurements were carried out on a nanometer scale on Pd-rich and Pd-free areas (see Figure S1). The recorded EELS spectra are indistinguishable in the region around 200 eV, corresponding to the expected value of the Cl L-edge, which unquestionably rules out the presence of chlorine in the palladated adducts with 2D BP.

**Figure 3:**
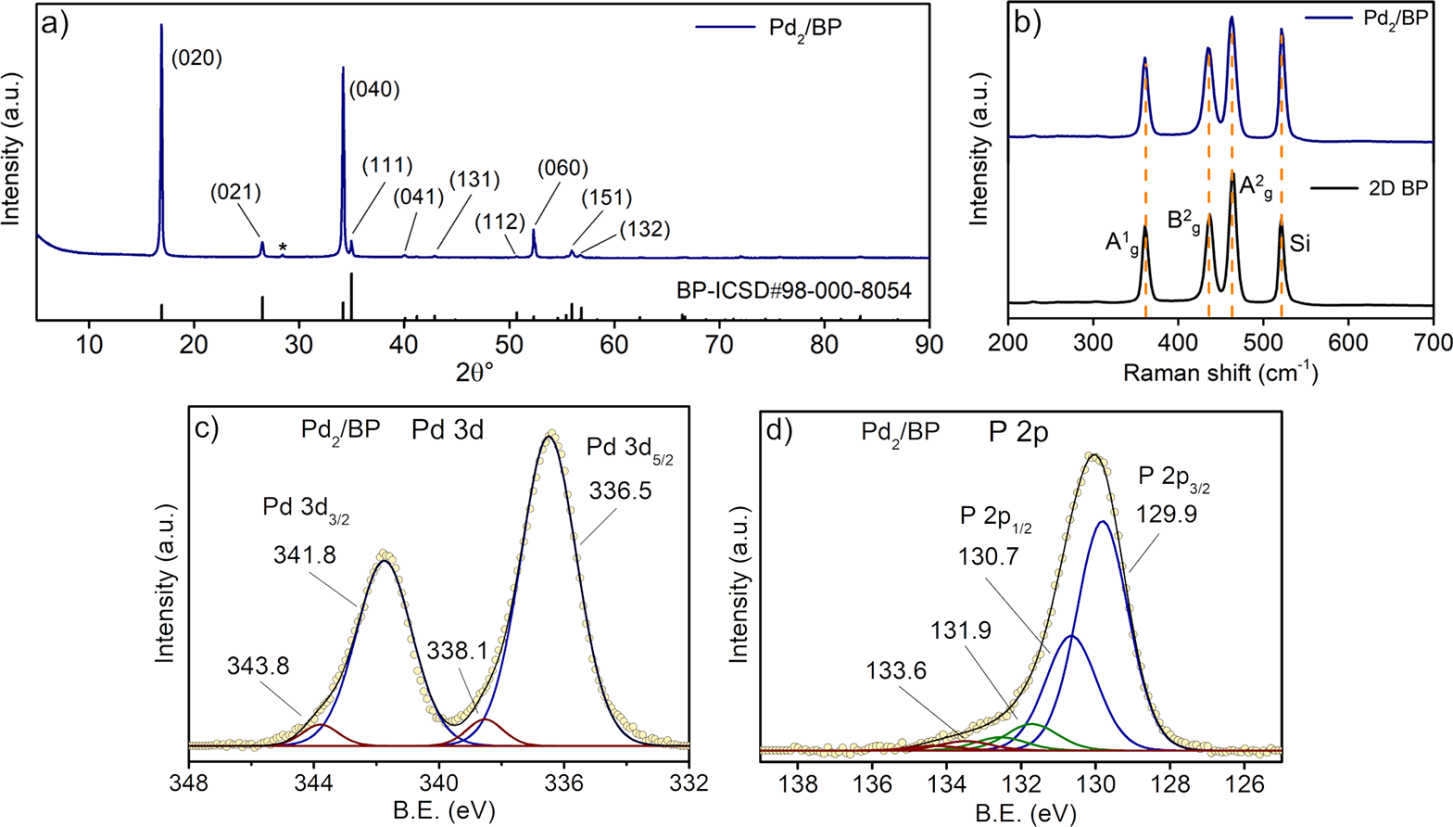
Spectroscopic characterization of Pd_2_/BP 3%. (a) Powder XRD spectrum. The reference pattern at the bottom corresponds to orthorhombic BP. The peak marked by an asterisk is a sample holder impurity. (b) Average Raman spectra of the functionalized material (top) and pristine 2D BP (bottom). Core level Pd 3d (c) and P 2p (d) XPS spectra.

Since electron microscopy provides information on the local structure of the sample under investigation, to firmly exclude the presence of nanoparticles and further assess the integrity of the BP lattice, bulk techniques were also used. The powder X-ray diffraction (XRD) spectrum of Pd_2_/BP (Figure 3a) features the typical pattern of pristine 2D BP with intense (0*k*0) reflections as an effect of preferential orientation in the sample. In detail, the three main peaks located at 2θ° = 16.9, 34.2, and 52.3° correspond to the (020), (040), and (060) reflections of BP, respectively, which suggests that BP retains its crystallinity after functionalization. Furthermore, no presence of additional phases could be observed in the XRD spectrum, in contrast to previously reported Pd NPs/BP.^26^ Raman spectroscopy agreed with XRD analysis. In particular, the Raman spectrum of Pd_2_/BP (Figure 3b), averaged within a set of several flakes to account for the polydispersity of the pristine material, features the three characteristic peaks at 360.7, 436.6, and 466.8 cm^–1^, corresponding to the A^1^_g_, B_2g_, and A^2^_g_ phonon modes of exfoliated BP, respectively. No relevant frequency shifts were observed in comparison to pristine BP (see also Figure S2).

To probe the electronic state of phosphorus and palladium in the material, XPS measurements were carried out at the Pd 3d and P 2p core levels. In the Pd 3d spectrum shown in Figure 3c, a dominant spin–orbit component is present, with Pd 3d_5/2_ = 336.5 eV. In comparison with the precursor **1** (Figure S3), the 3d_5/2_ component of Pd_2_/BP 3% is shifted to lower binding energy (BE) by 0.7 eV, suggesting a more reduced oxidation state of Pd in Pd_2_/BP. The observed BE value is closer to that of bulk metallic Pd(0)^47^ than to those typical of Pd(II) salts (see also Table S1), though it is clearly distinguishable from both these extremes. An additional and more oxidized Pd species (brown line in Figure 3c) is also present, with Pd 3d_5/2_ = 338.1 eV, amounting to ca. 5% of the whole Pd. This minor component is higher in BE in comparison to the starting complex **1** and can be reasonably accounted for with some oxidation of the main Pd(0)-like species, a common feature in the XPS spectra of Pd(0) systems.^48,49^ Notably, increasing the Pd loading from 3% to 6% had no effect on the Pd 3d spectrum of Pd_2_/BP within the experimental error (see Figure S4a), suggesting an equivalent chemical state of Pd in the two samples. The P 2p core level spectrum in Figure 3d features the two intense peaks of pristine BP at 129.9 and 130.7 eV, corresponding to P 2p_3/2_ and P 2p_1/2_, respectively (see also Figure S5). In addition, two components are present shifted to higher binding energies, with P 2p_3/2_ and P 2p_1/2_ at 131.9 and 133.6 eV (with a small variability, within 0.3 eV, depending on the measured sample), respectively. The latter, shown by the brown line in Figure 3d, is assigned to PO_*x*_ species, while the former, shown by a green line and closer to BP peaks, is attributed to P–Pd. This interpretation is strengthened by air exposure studies (see Figure S6), showing that only the high-energy component grows after 12 h of air exposure. Since, in contrast to previously reported MP_*x*_–BP heterostructures (M = metal),^50,51^ no peaks were observed at BE values lower than that of pristine BP, PdP_*x*_ phases could be firmly ruled out. Furthermore, the core level Cl 2p XPS spectrum of Pd_2_/BP (Figure S7) confirmed the absence of chlorine. This last finding, established via EDX, EELS, and XPS, strongly questions the presence of the allylic moiety as well. To further investigate this fundamental point, ^13^C MAS (magic angle spinning) NMR measurements were carried out on 2D BP reacted with 1* (^13^C-labeled **1**), prepared by starting from 1-^13^*C*-allyl alcohol as described in the Supporting Information. Interestingly, no signal consistent with the isotopically enriched allyl ligand was detected in the spectra between 30 and 140 ppm, definitely ruling out the functionalization of 2D BP with Pd–allyl. The only observed spectral feature was a broad signal in the 30–50 ppm region having a very low signal to noise ratio (Figure S8a). Since substantially the same spectrum was also obtained for 2D BP and Pd_2_/BP 6% (Figure S8a), this signal can be reasonably ascribed to minor amounts of alkylated species bound to 2D BP, accidentally formed by a reaction with the solvents during the exfoliation process (further details are reported in the Supporting Information).

**Figure 4:**
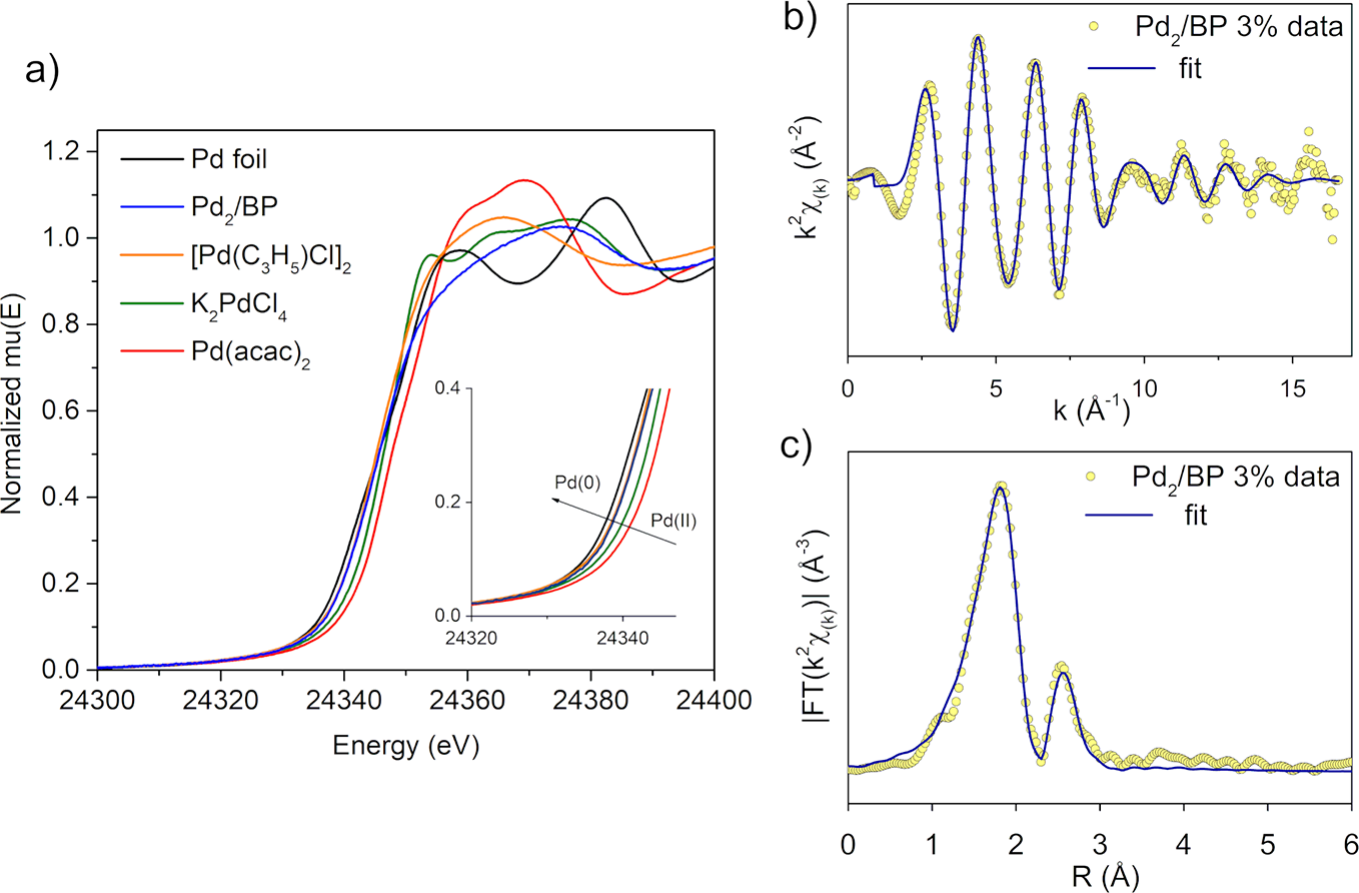
XAS characterization of Pd_2_/BP 3% at the Pd K-edge. (a) Normalized XANES spectra of Pd_2_/BP and Pd reference materials. (b) EXAFS *k*^2^-weighed spectrum of Pd_2_/BP and (c) magnitude of its Fourier transform. Dots are experimental data; continuous lines correspond to the best calculated fit.

Since elemental analysis and ^13^C NMR spectroscopy together ruled out the permanence of both chloride and allyl ligands in the coordination sphere of Pd, it is likely that **1** has undergone a reductive elimination of allyl chloride upon interaction with 2D BP. The problem then arises to infer the actual bonding situation of the Pd sites in the functionalized material. Remarkably, XPS pointed to a well-defined Pd environment. Since Pd aggregates were firmly excluded, interlayer structures should be considered to account for the high concentration of the metal, with Pd atoms lying amidst two phosphorene layers. Indeed, BP intercalation compounds have been reported for alkali metals, namely Li, Na, K, Rb, and Cs,^52−54^ though these compounds are better described as being formed by a reduced BP^–^ lattice with intercalated M^+^ ions. In 2016 Özyilmaz et al. reported the doping of a BP flake with Cu atoms via atomic layer deposition (ALD)^55^ and showed with DFT calculations that single interlayer Cu atoms, alongside surface adatoms, are a possible outcome of the ALD process. To gain insights into the Pd coordination shell in our system, XAS measurements were carried out at the Pd K-edge. Figure 4a shows the XANES (X-ray absorption near edge structure) spectra of Pd_2_/BP 3%, in comparison with some reference materials. The relative positions of the rising edges confirms that the oxidation state of Pd in Pd_2_/BP is closer to Pd(0) than to Pd(II), in agreement with XPS findings. However, in view of the low XANES energy resolution at the Pd K-edge (about 6 eV^56^), as well as its dependence on the coordination geometry, an accurate distinction between the chemical state of Pd in Pd_2_/BP and **1** was prevented, in contrast to XPS (see Table S1). The EXAFS (extended X-ray absorption fine structure) *k*^2^-weighed spectrum and the corresponding Fourier transform of Pd_2_/BP 3% are reported in Figure 4b,c, respectively. Regardless of the metal loading (see also Figure S10), the FT spectrum of Pd_2_/BP shows a first-shell coordination just below *R* = 2 Å, which could be fitted using Pd–P bonds, and a second-shell peak below *R* = 3 Å, safely assigned to Pd–Pd scattering. The accurate bond distances obtained after data fitting and phase correction for Pd_2_/BP and **1** are reported in Table 1. The structural parameters of various reference materials are also shown for comparison. Notably, Pd_2_/BP 3% and 6% look identical in XAS analysis, suggesting that the coordination sphere of Pd is the same in the two samples, in nice agreement with XPS evidence.

**Table 1:** Interatomic Distances and Coordination Numbers Extracted from EXAFS Data Analysis^a^

sample	path	CN	*r* (Å)	σ^2^ (Å^2^)
**Pd**_**2**_**/BP 3%**	Pd–P	2.8(2)	2.34(1)	0.0076(7)
	Pd–Pd	0.8(2)	2.82(1)	0.011(2)
**Pd**_**2**_**/BP 6%**	Pd–P	2.7(2)	2.34(1)	0.0078(7)
	Pd–Pd	1.1(3)	2.83(1)	0.012(2)
Pd NPs/BP^b^	Pd–P	1.7(6)	2.26(3)	0.0018(6)
	Pd–Pd	8(2)	2.73(2)	0.0016(4)
Pd foil^b^	Pd–Pd	12	2.74(1)	0.0059(4)

a Values in parentheses represent the error on the last digit.

b Data from ref (26).

The measured Pd–P distance of 2.34(1) Å in Pd_2_/BP suggests a quite strong interaction between Pd and BP, consistent with the proven ability of BP to take part in coordinative bonds. Remarkably, the second shell of Pd features a Pd–Pd distance of 2.82(1) Å, appreciably larger in comparison to metallic Pd(0) (2.751 Å),^57^ previously measured by some of us in bulk Pd foil (2.74 ± 0.01 Å) and Pd NPs/BP (2.73 ± 0.02 Å).^26^ This discrepancy allows the presence of Pd NPs in Pd_2_/BP to be ultimately ruled out, in agreement with all the other techniques, particularly HAADF-STEM and XPS. The Pd–P distance of 2.34(1) Å in Pd_2_/BP was significantly elongated in comparison to the value of 2.26(3) Å found in Pd(0) NPs/BP.^26^ Moreover, the observed Pd–Pd separation (2.82 Å) does not agree with the homologous distance determined in either PdP_2_ (3.10 Å)^58^ or PdP_3_ (3.85 Å),^59^ thus excluding once more the formation of Pd phosphide aggregates. The obtained coordination numbers (CNs) associated with these bonds are extremely valuable to infer a coherent structural model. Remarkably, Pd–P and Pd–Pd CNs have almost integer values of 3 and 1, respectively, nicely reproduced in the two samples Pd_2_/BP 3% and 6%. This finding points to the existence of a well-defined Pd environment, in which every Pd atom is bonded to three P atoms, with an average Pd–P distance of 2.34(1) Å, and to a second Pd center at 2.82(1) Å.

Since the overall integrity of the BP lattice is preserved after functionalization, as pointed out experimentally, the candidate structure of Pd_2_/BP should exhibit only slight distortions with respect to pristine 2D BP. Different structural models featuring a Pd_2_ dimer sandwiched between two phosphorene layers were optimized by computational analysis. Obviously, in view of the maintenance of the phosphorene lattice, the Pd_2_ unit must lie parallel to the channel, since an orthogonal arrangement would cause a severe elongation of the interlayer distance, which contrasts with the experimental evidence. A computational analysis also ruled out the potential localization of the Pd_2_ units on top of the BP surface (see Figure S11), in view of disfavoring structural and energetic features, being less stable than the intercalated Pd_2_ units by at least +35.0 kcal mol^–1^.

**Figure 5:**
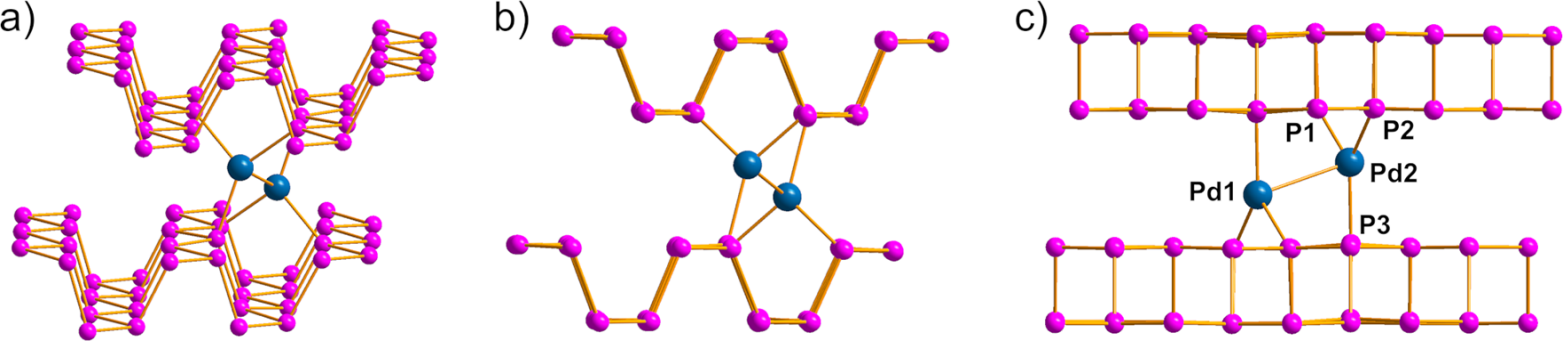
(a) DFT optimized model of Pd_2_/BP featuring a trigonal-planar ligand geometry around Pd. Different views of the same model along the zigzag (b) and armchair (c) directions are shown. Interatomic distances (Å) in (c): Pd1–Pd2 = 3.015; Pd2–P1 = 2.372; Pd2–P2 = 2.345; Pd2–P3 = 2.367.

Different isomers were obtained with energy variations of less than 2 kcal mol^–1^, suggesting a substantial flatness of the potential energy surface (PES) associated with the hosting of Pd_2_ between two layers. All of the isomers feature a Pd–Pd distance in the range 2.8–3.0 Å, Pd–P distances of 2.3 Å, and Pd–P coordination numbers between 3 and 4, in fair agreement with XAS structural parameters. For the sake of clarity, Figure 5 shows one of the most stable optimized isomers, with a Pd1–Pd2 distance of 3.01 Å and a trigonal-planar coordination of phosphorus around each metal center, typically associated with Pd(0), with a staggered conformation of the Pd–P bonds. Notably, such an arrangement does not significantly perturb the lattice of phosphorene, the interlayer distance being elongated by ca. 0.55 Å. A flatness of the PES with respect to the metal–metal distance is not completely unknown, and a previous experimental and computational investigation^60−62^ highlighted a similar behavior for some Pt clusters, in which large variations of the Pt–Pt bonds were induced by small changes in the chemical conditions, such as the nature of the crystallization solvent. Remarkably, a related arrangement of Pd_2_ units sitting amidst layers of carbon nitride was recently described by Pérez-Ramírez et al., featuring a broad range of structural isomers associated with the Pd–Pd distance.^43^

Indirect confirmatory evidence for the low accessibility of the sandwiched Pd_2_ units has been acquired experimentally by testing the catalytic performance of Pd_2_/BP in the hydrogenation of unsaturated organic substrates, such as 1-octene and phenylacetylene (see the Supporting Information). In agreement with the inaccessibility of the metal centers, no catalytic activity was observed with Pd_2_/BP despite Pd-based systems being usually very active in these processes.^63−69^

**Figure 6:**
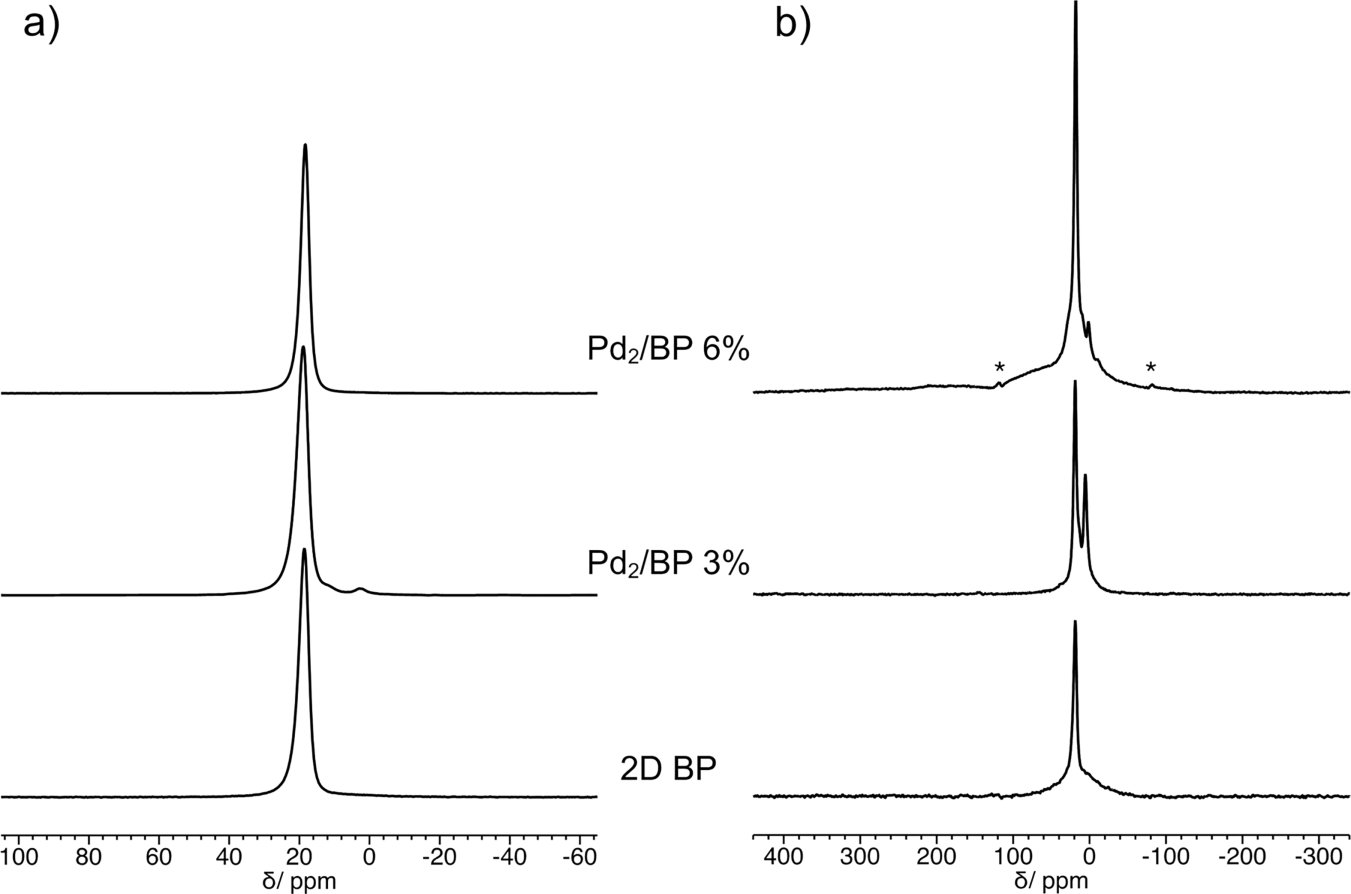
^31^P MAS NMR spectra of 2D BP, Pd_2_/BP 3%, and Pd_2_/BP 6%, recorded at a MAS frequency of 20 kHz, using the direct excitation (DE) pulse sequence with a recycle delay between consecutive transients of (a) 200 s (quantitative spectra) and (b) 0.2 s (selective spectra). Asterisks indicate spinning sidebands.

In order to further characterize the Pd–P binding, ^31^P MAS NMR spectra were recorded on both Pd_2_/BP 3% and 6% and compared with that of pristine 2D BP. The signal of ^31^P nuclei bonded and/or in proximity to one or two Pd atoms, in addition to exhibiting a different chemical shift, should show a complex shape due to the effects of direct dipolar and indirect (*J*) couplings with ^105^Pd nuclei. ^105^Pd, the only isotope of Pd with nonzero spin, is a nucleus with 22.3% natural abundance, spin 5/2, and a sizable quadrupolar moment.^70^^31^P MAS NMR spectra generally show a multiplicity of lines arising from *J* and residual (not averaged out by MAS) dipolar couplings with ^105^Pd.^71^ In Pd_2_/BP several situations might occur for those ^31^P nuclei bonded and/or spatially close to Pd atoms depending on the Pd isotope distribution, the number of bonds, and the distance between P and Pd atoms, ultimately leading to a composite signal with multiple and broad components.^72^ Moreover, the interaction with ^105^Pd is expected to significantly shorten the ^31^P spin–lattice relaxation time. Surprisingly, for Pd_2_/BP 3% and 6% the ^31^P MAS spectra acquired under quantitative conditions (Figure 6a) substantially show only an intense and slightly asymmetric peak typical of bulk and exfoliated BP^73−75^ (only for Pd_2_/BP 3% additional weak resonances are observed at 11.5 and 2.4 ppm, accounting for about 1.5% of the whole spectral intensity, arising from products of accidental BP oxidation, i.e. variously protonated PO_4_^3–^ and HPO_3_^2–^ groups, respectively^73,76^). It must be observed that, while in these spectra signals attributable to P atoms bonded to Pd are not distinguished, the chemical shift of BP in Pd_2_/BP 6% is slightly lower than that in 2D BP and Pd_2_/BP 3% (18.3 vs 18.8 ppm). On the other hand, selective ^31^P MAS NMR spectra, recorded with short recycle delays for highlighting signals from faster-relaxing ^31^P nuclei (Figure 6b and Figure S8d), show a weak shoulder at about 38 ppm for Pd_2_/BP 3% and a complex signal covering a wide frequency range for Pd_2_/BP 6% (see an example in Figure 6b). The latter signal can be phenomenologically described as a superposition of a peak at 26 ppm, a broad peak at 33 ppm, and weak bumps at higher frequencies (Figure S8g). This composite signal, also investigated at variable MAS frequency and temperature (Figure S8e,f), is ascribable to ^31^P nuclei interacting with ^105^Pd nuclei in the complex spin system of Pd_2_/BP. Moreover, its contribution to the quantitative spectrum of Pd_2_/BP 6% can be estimated to be about 3%, in good agreement with the Pd content, the Pd_2_/BP hypothesized structure, and the ^105^Pd natural abundance. When the broadness and the expected very low intensity are taken into account, the lack of a clear observation of the same signal in the spectrum of Pd_2_/BP 3% is not surprising. All this considered, it can be inferred that the signal of P atoms interacting with zero-spin Pd nuclei underlies the main signal of BP, likely determining its different chemical shift in Pd_2_/BP 6%.

### 2.2 Electrocatalytic Studies

Black phosphorus, as a 2D semiconductor, has received much attention for its application in energy conversion,^77^ including electrochemical energy storage and electrocatalysis.^78,79^ Notably, 2D BP drop-casted on a glassy-carbon electrode (GCE) was shown to promote the HER, though pristine 2D BP does not behave as an efficient catalyst, its performance being highly affected by the morphology and dimensions of the flakes.^80,81^ Pd_2_/BP was tested to see whether the interlayer coordination of Pd_2_ units could be a way to enhance the HER activity of 2D BP. The catalyst evaluation was carried out using a three-electrode cell with a rotating-disk working electrode (RDE), a commercial Ag/AgCl reference electrode, and Au gauze as a counter electrode. The catalyst material was drop-casted above the glassy-carbon (GC) surface of the RDE, and then a thin Nafion film was applied with a 0.5%_w_ Nafion solution in 2-propanol to ensure a better adhesion to the GC surface. As shown in Figure S12, our 2D BP has a poor activity for the HER in 0.5 M H_2_SO_4_. The reaction *E*_onset_ is −0.13 V vs RHE, and the maximum current density recorded at −0.6 V vs RHE is about −500 μA cm^–2^.

**Figure 7:**
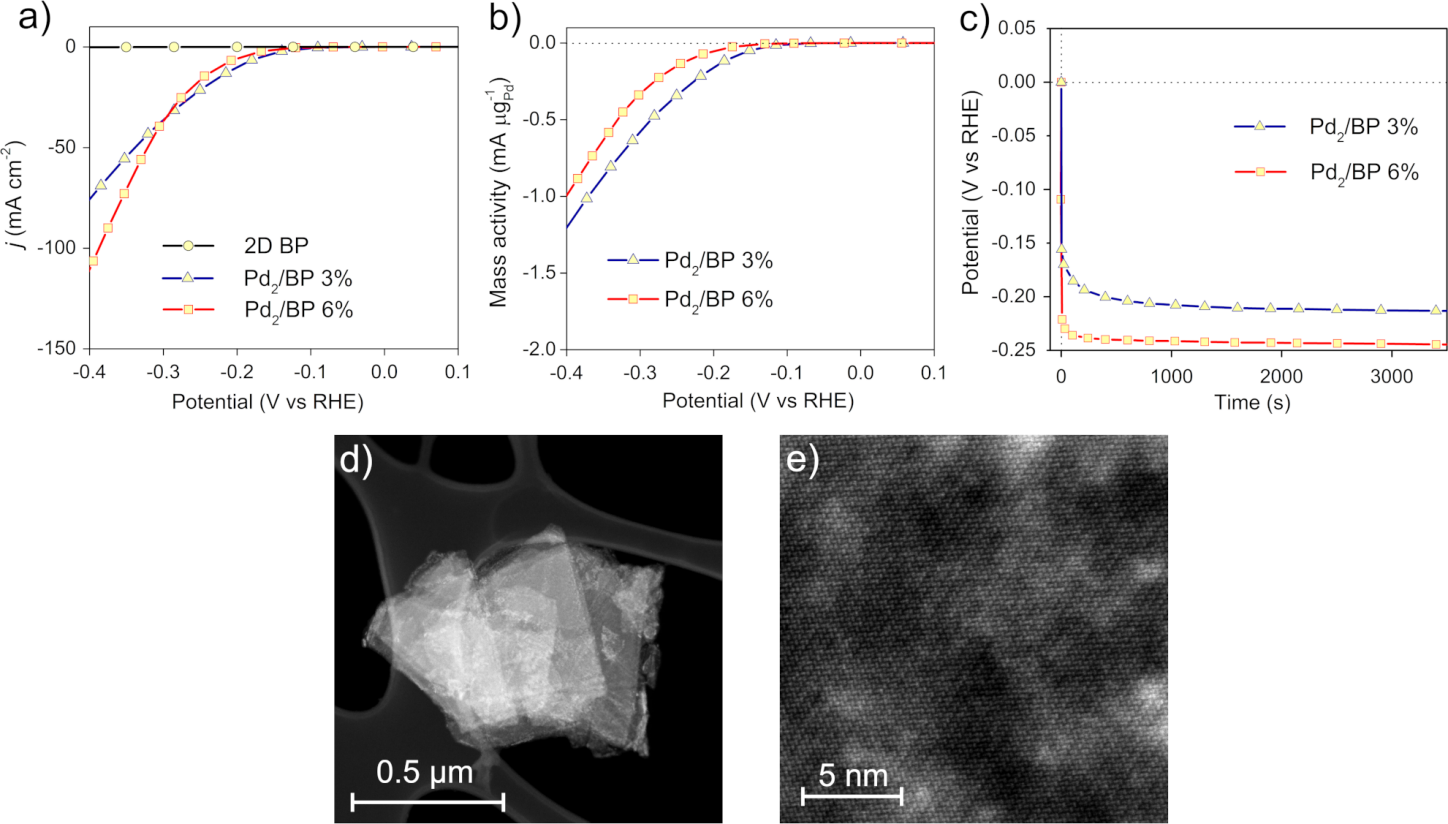
Electrocatalytic activity of 2D BP and Pd_2_/BP in HER from 0.5 M H_2_SO_4_. (a) Comparison of the linear sweep voltammetry (LSV) activity of 2D BP, Pd_2_/BP 3%, and Pd_2_/BP 6% (scan rate 1 mV s^–1^, 1600 rpm RDE rotations). (b) LSV normalized to the Pd content. (c) Chronopotentiometryat −1 mA (−5 mA cm^–2^) for 3600 s (1600 rpm RDE rotations). (d) Flakes of Pd_2_/BP 3% exhaust catalyst recovered after chronoamperometric measurements, drop-casted on a carbon grid. (e) High-resolution HAADF-STEM micrograph taken from the flake in (d).

In contrast, Pd_2_/BP shows a superior activity for HER in comparison to pristine 2D BP, as shown in Figure 7a. Both Pd_2_/BP 3% and 6% have the onset potential *E*_onset_ = −0.1 V vs RHE, similar to that recorded for 2D BP, but these samples reach current densities one order of magnitude higher than that of the pristine material, namely −110 mA cm^–2^ at 0.4 V vs RHE and −75 mA cm^–2^ at −0.4 V vs RHE for the 6% and 3% catalysts, respectively. Thus, the presence of discrete interlayer Pd_2_ units has an active role in promoting the hydrogen evolution reaction.

Since the metal loading is related to the current density recorded during the measurements, the LSV (linear sweep voltammetry) voltammograms in Figure 7b were normalized to the palladium content of each catalyst (mass activity). Upon normalization, the two catalysts Pd_2_/BP 3% and 6% show very similar activities, suggesting that the HER is limited solely by the number of Pd active sites on the catalyst. This observation provides an indirect confirmation of their structural analogy, in accordance with previous characterizations.

The catalyst stability during hydrogen evolution was investigated through galvanostatic experiments, applying to the working electrode a constant current load of −1 mA (5 mA cm^–2^) for 3600 s. As reported in Figure 7c, Pd_2_/BP is stable during 1 h of electrolysis, with no electrochemical evidence of catalyst alteration under working conditions. To better assess this point, the exhaust Pd_2_/BP 3% catalyst was recovered by cleaning the working electrode in 2-propanol with ultrasound and its morphology was studied via TEM and HAADF-STEM. As it turned out, the catalytic process does not affect the overall morphology of the material (Figure 7d,e and Figures S13 and S14), which still features intact flakes with Pd homogeneously dispersed (see also the EDS map in Figure S13) and the absence of metal aggregates, as also revealed by high-resolution imaging, in nice agreement with the electrochemical evidence. In addition, an ICP-AES analysis of the exhaust solution recovered after the galvanostatic experiment confirmed the absence of Pd leaching and the catalyst stability for promoting the HER at a constant current load of −1 mA.

In order to preliminarily investigate the catalyst durability, two consecutive sets of 90 cyclic voltammetry (CV) scans were performed between 0 and −0.325 V vs RHE at a scan rate of 20 mV s^–1^ (Figure S15). Moreover, the Pd_2_/BP 6% reusability was investigated by recovering the electrode after the first set of CV scans; the electrode was washed with distilled water, dried, and stored in air for 3 h before performing the second set of CV scans. A negligible current density drop of 11 mA cm^–2^ among 180 CV scans was recorded (8 mA cm^–2^ in the first batch, Figure S15a, and 3 mA cm^–2^ in the second batch, Figure S15b); thus no massive catalyst decomposition occurs during the accelerated durability test. In addition, the unchanged catalyst activity observed between the 90th (first batch of CVs, Figure S15a) and the 91st cycle (second batch of CVs, Figure S15b) highlighted the strong stability of Pd_2_/BP 6% and adhesion to the glassy-carbon electrode, which are two important features for making the catalyst recyclable in principle and therefore useful for assembling real electrolysis cells.

## 3 Conclusions

In summary, we have successfully decomposed the organopalladium(II) complex **1** in the presence of 2D BP to provide BP flakes functionalized with rare discrete Pd–Pd units, using a mild synthetic protocol. A variety of solid-state characterization techniques such as EXAFS, HAADF-STEM, XPS, and NMR spectroscopy have been used to ascertain the structure of Pd_2_ sites. In particular, EXAFS investigations, backed up by DFT modeling, were crucial to highlight an unprecedented *interlayer* coordination of Pd_2_, sandwiched between stacked BP layers. Remarkably, the BP lattice retains its overall integrity upon functionalization, while phosphorus atoms efficiently stabilize the Pd_2_ units, preventing nanoparticle formation. This study represents the first full structural elucidation of low-nuclearity metal sites in functionalized BP. A preliminary electrochemical study confirmed a notably higher activity of Pd_2_/BP in the HER from acidic medium in comparison to pristine 2D BP. Further studies aimed at exploring the reactivity of the dipallada units and their possible replacement by other transition metals are in progress.

## Supporting Information Available

Supplementary figures and experimental methods, including all of the syntheses and instrumental techniques (PDF)
