# Supplementary material for: Interlayer Coordination of Pd–Pd Units in Exfoliated Black Phosphorus
Source: J Am Chem Soc. 2021 Jun 29;143(27):10088–98. doi: 10.1021/jacs.1c01754 (PMC9295127; doi:10.1021/jacs.1c01754)
Supplement: Supplementary file 1 — ja1c01754_si_001.pdf [file ja1c01754_si_001.pdf]

# Interlayer Coordination of Pd–Pd Units in Exfoliated Black Phosphorus

Matteo Vanni,[a,b]* Marco Bellini,[a] Silvia Borsacchi,[c,d] Lucia Calucci,[c,d] Maria Caporali,[a]* Stefano Caporali,[e] Francesco D'Acapito,[f] Marco Geppi,[d,g] Andrea Giaccherini,[h] Andrea Ienco,[a] Gabriele Manca,[a]* Antonio Massimiliano Mio,[i] Giuseppe Nicotra,[i] Werner Oberhauser,[a] Manuel Serrano-Ruiz,[a] Martina Banchelli,[j] Francesco Vizza,[a] Maurizio Peruzzini[a]*

[a]Institute for the Chemistry of Organometallic Compounds (CNR-ICCOM), Via Madonna del Piano 10, 50019 Sesto Fiorentino, Italy

[b]Department of Biotechnology, Chemistry and Pharmacy, University of Siena, 53100 Siena, Italy

[c]Institute for the Chemistry of Organometallic Compounds (CNR-ICCOM), SS Pisa, Via Moruzzi 1, 56124 Pisa, Italy

[d]Center for Instrument Sharing of the University of Pisa (CISUP), Lungarno Pacinotti 43/44, 56126 Pisa, Italy

[e]Department of Industrial Engineering, University of Florence, Via di S. Marta 3, 50139 Firenze, Italy

[f]CNR-IOM-OGG, c/o European Synchrotron Radiation Facility, 71 Avenue des Martyrs, CS 40220, 38043 Grenoble, Cedex 9 France

[g]Department of Chemistry and Industrial Chemistry (DCCI), University of Pisa, Via Moruzzi 13, 56121 Pisa, Italy

[h]Department of Earth Sciences, University of Florence, Via La Pira 4, 50121 Firenze, Italy

[i]Institute for Microelectronics and Microsystems (CNR-IMM), VIII strada 5, I-95121 Catania, Italy

[j]Institute of Applied Physics 'Nello Carrara' (CNR-IFAC), Via Madonna del Piano 10, 50019 Sesto Fiorentino, Italy

**Corresponding Authors**

*E-mail: maurizio.peruzzini@iccom.cnr.it

*E-mail: maria.caporali@iccom.cnr.it

*E-mail: gmanca@iccom.cnr.it

*E-mail: matteo.vanni@iccom.cnr.it

# 1. Supplementary Figures and Tables

Figure S1. EELS spectra
Figure S2. Raman statistics
Figure S3. Pd 3d XPS spectrum of **1**
Table S1. Pd 3d XPS data
Figure S4. Pd 3d and P 2p XPS spectra of $Pd_2$/BP 6%
Figure S5. P 2p XPS spectrum of pristine 2D BP
Figure S6. Time evolution of the P 2p XPS spectrum of $Pd_2$/BP 6%
Figure S7. Survey XPS spectrum and Cl 2p core level XPS spectrum of $Pd_2$/BP 6%
Figure S8. Solid state MAS NMR spectra
Figure S9. ATR-FTIR spectra
Figure S10. EXAFS spectrum of $Pd_2$/BP 6%
Figure S11. DFT model of $Pd_2$ adsorbed above bilayer BP
Figure S12. LSV of 2D-BP
Figure S13. HAADF-STEM characterization of $Pd_2$/BP post-catalysis
Figure S14. TEM characterization of $Pd_2$/BP post-catalysis
Figure S15. CVs of $Pd_2$/BP 6%
Figure S16. Ambient stability study
Figure S17. Additional HAADF-STEM images of $Pd_2$/BP 6%
Figure S18. Powder XRD spectrum of $Pd_2$/BP 6%
Figure S19. EXAFS spectrum of **1**

# 2. Synthesis and catalysis

2.1 General methods and materials
2.2 BP synthesis and exfoliation
2.3. Synthesis of $[Pd(C_3H_5)Cl]_2$ (**1**)
2.4 Synthesis of $[Pd(1\text{-}^{13}C\text{-}C_3H_5)Cl]_2$ (**1***)
2.5 2D BP functionalization with **1**
2.6 2D BP functionalization with **1***
2.7 Hydrogenation of phenylacetylene
2.8 Hydrogenation of 1-octene
2.9 Electrocatalytic measurements

# 3. Characterization of the material

3.1 Inductively coupled plasma – atomic emission spectroscopy (ICP-AES)
3.2 TEM microscopy
3.3 SEM microscopy

3.4 HAADF-STEM microscopy, EDS and EELS spectroscopy
3.5 Powder X-Ray Diffraction (XRD)
3.6 Raman scattering
3.7 ATR-FTIR
3.8 X-ray Photoelectron Spectroscopy (XPS)
3.9 Solid State NMR Spectroscopy
3.10 X-Ray Absorption Spectroscopy (XAS)
3.11 GC-MS analysis
3.12 DFT calculations

# 1. Synthesis and Catalysis

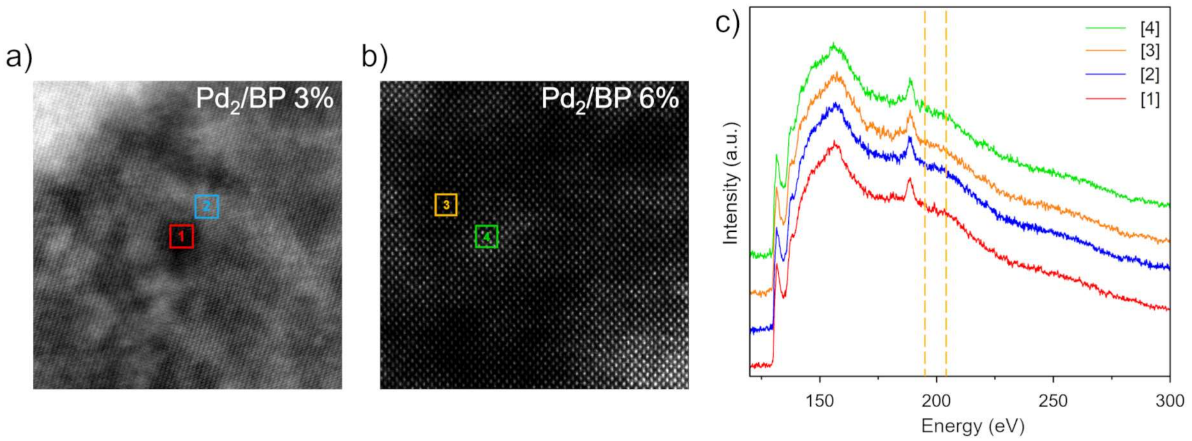


**Figure S1.** Comparative EELS nanoanalysis of $Pd_2$/BP 3% (a) and $Pd_2$/BP 6% (b). (c) EELS spectra acquired in Pd-rich regions (highlighted areas 2 and 4) and Pd-poor regions (highlighted areas 1 and 3). EELS spectra in (c) have been shifted on the vertical scale for the sake of comparison. The region around 200 eV highlighted with vertical bars comprises the Cl L-edge (no modifications observed).

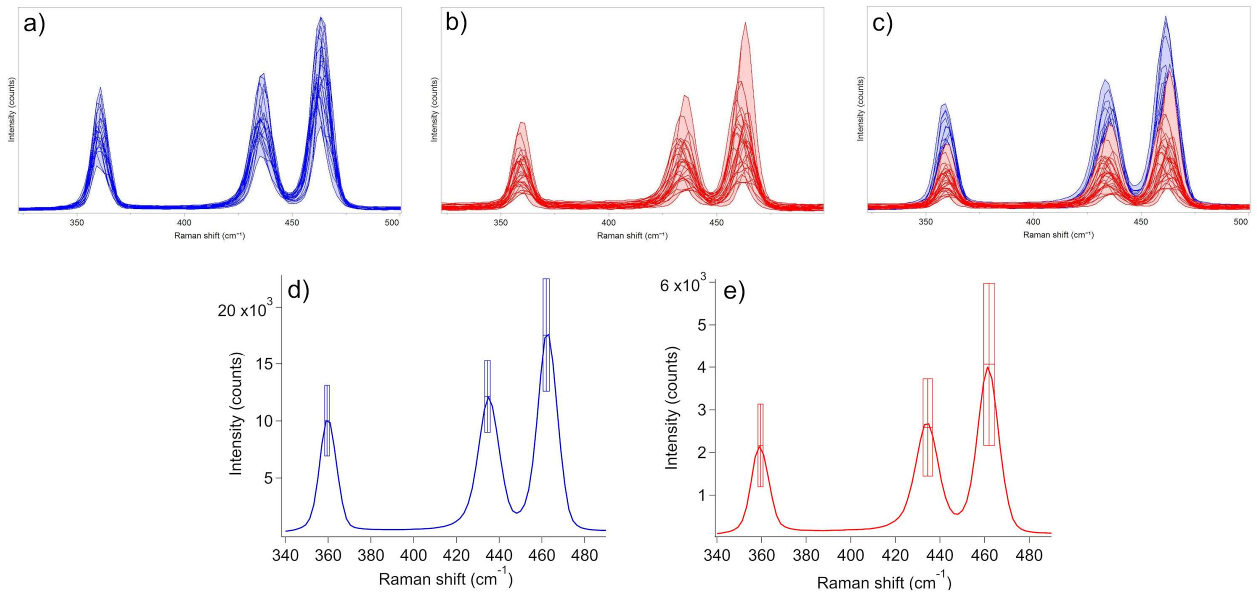


**Figure S2.** Statistical Raman analysis of $Pd_2$/BP 6% and pristine BP based on 60 measurement points; the spectra are normalized to the silicon band. Ensemble of Raman mapping spectra of $Pd_2$/BP 6% (a) and pristine BP (b) and their superposition (c). Intensity and Raman shift standard deviation of the Raman bands in $Pd_2$/BP 6% (d) and pristine BP (e). Though a larger dispersion was observed for each phonon mode in $Pd_2$/BP compared to pristine BP, on average no frequency shift was observed.

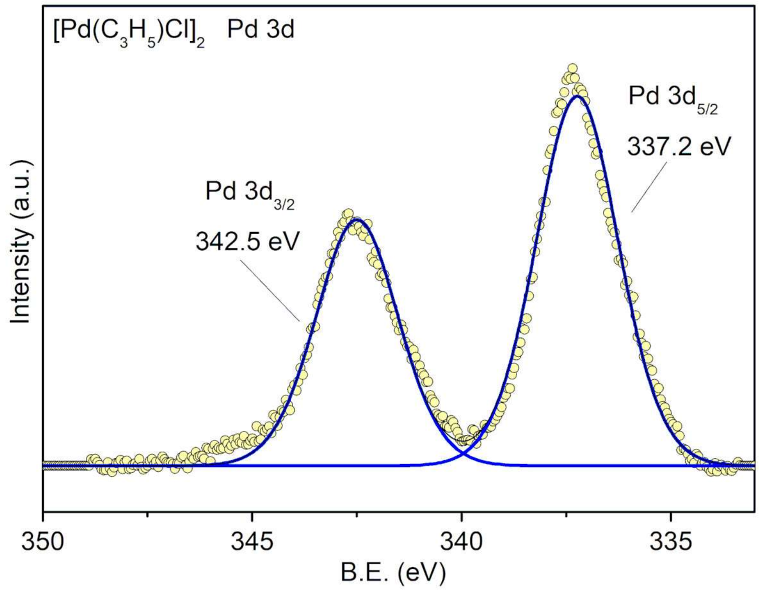


**Figure S3.** Core level Pd 3d XPS spectrum of **1**.

**Table S1. Pd 3d XPS binding energies.**

| Sample | Pd $3d_{5/2}$ (eV) | Pd $3d_{3/2}$ (eV) |
|---|---|---|
| $Pd(acac)_2$ | 340.8 | 346.1 |
| $K_2PdCl_4$* | 338.2 | / |
| **1** | 337.2 | 342.5 |
| $Pd_2$/BP 3% | 336.5 | 341.8 |
| $Pd_2$/BP 6% | 336.7 | 342.0 |
| Pd NPs† | 335.8 | 341.0 |

* Data from reference (S1). † The reported data correspond to the metallic Pd(0) core of the NPs from reference (S2).

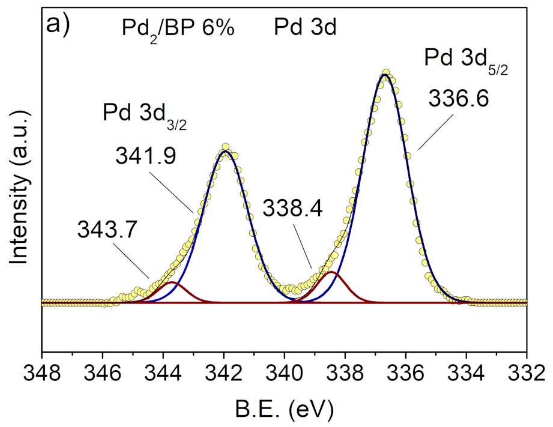


**Figure S4.** Core level Pd 3d (a) and P 2p (b) XPS spectra of $Pd_2$/BP 6%. In the P 2p deconvolution, the green component (P $2p_{3/2}$ = 132.2 eV) is attributed to the P–Pd interaction, whereas the higher energy component in red (P $2p_{3/2}$ = 133.9 eV) to surface $PO_x$ species resulting from oxidation. The red component (Pd–O) amount to about 7 % of the whole integrated area.

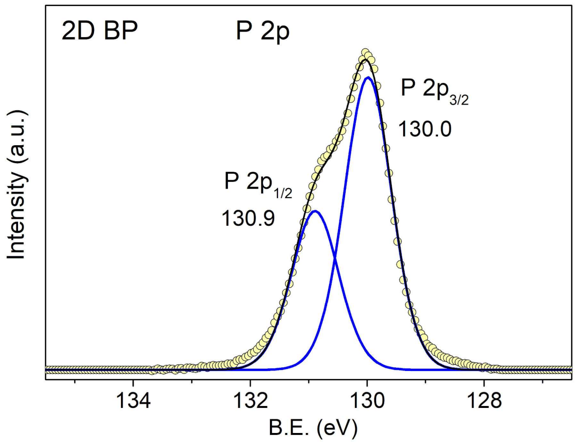


**Figure S5.** Core level P 2p XPS spectrum of pristine 2D BP.

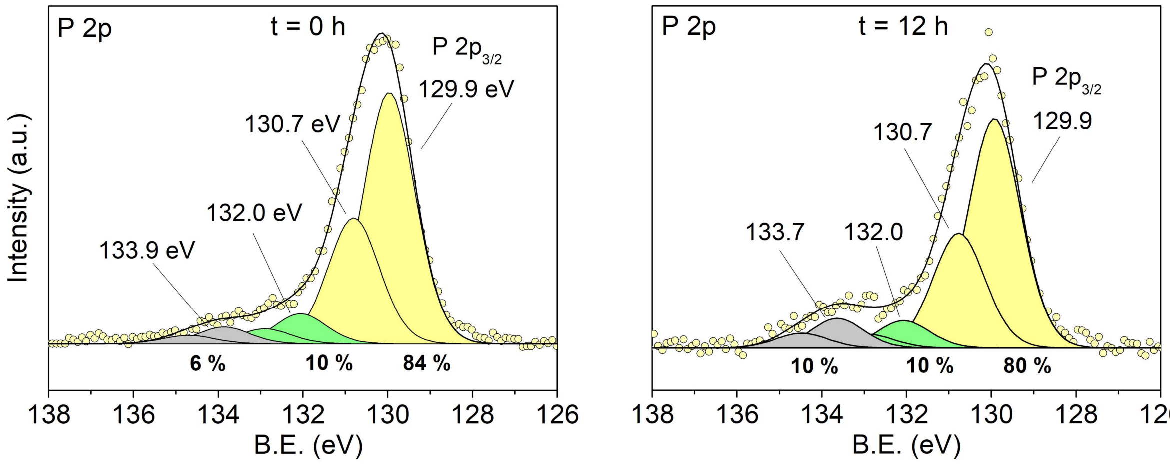


**Figure S6.** P 2p XPS spectrum of $Pd_2$/BP 6% freshly prepared (left) and after 12 h of air exposure. Percentages below the deconvoluted peaks represent the relative weight of each component. After 12 h, the more oxidized species in grey had increased at the expense of pristine BP (yellow component) from 6 % to 10 %. In contrast, the green component remained constant.

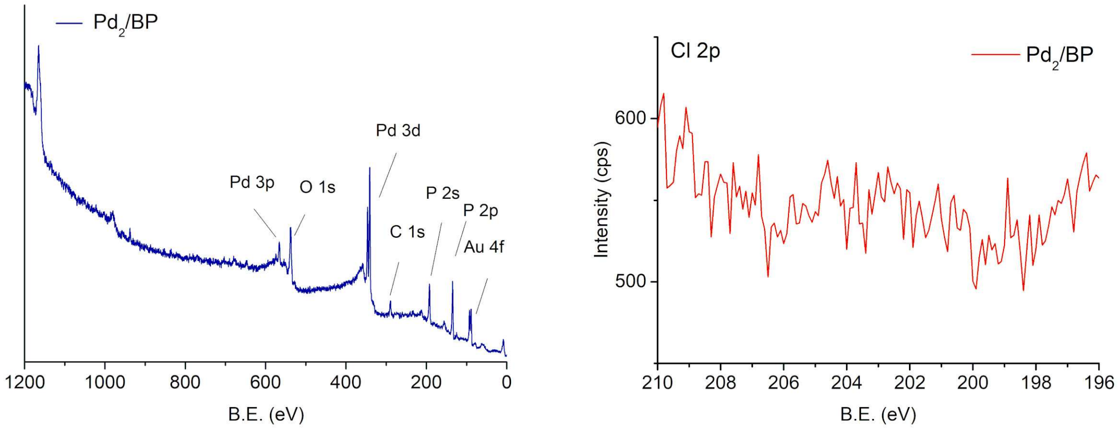


**Figure S7.** Survey XPS spectrum (left) and core level Cl 2p spectrum (right) of $Pd_2$/BP 6%. No chlorine was observed within the instrumental detection limit. The Au signal in the survey spectrum is due to an internal standard.

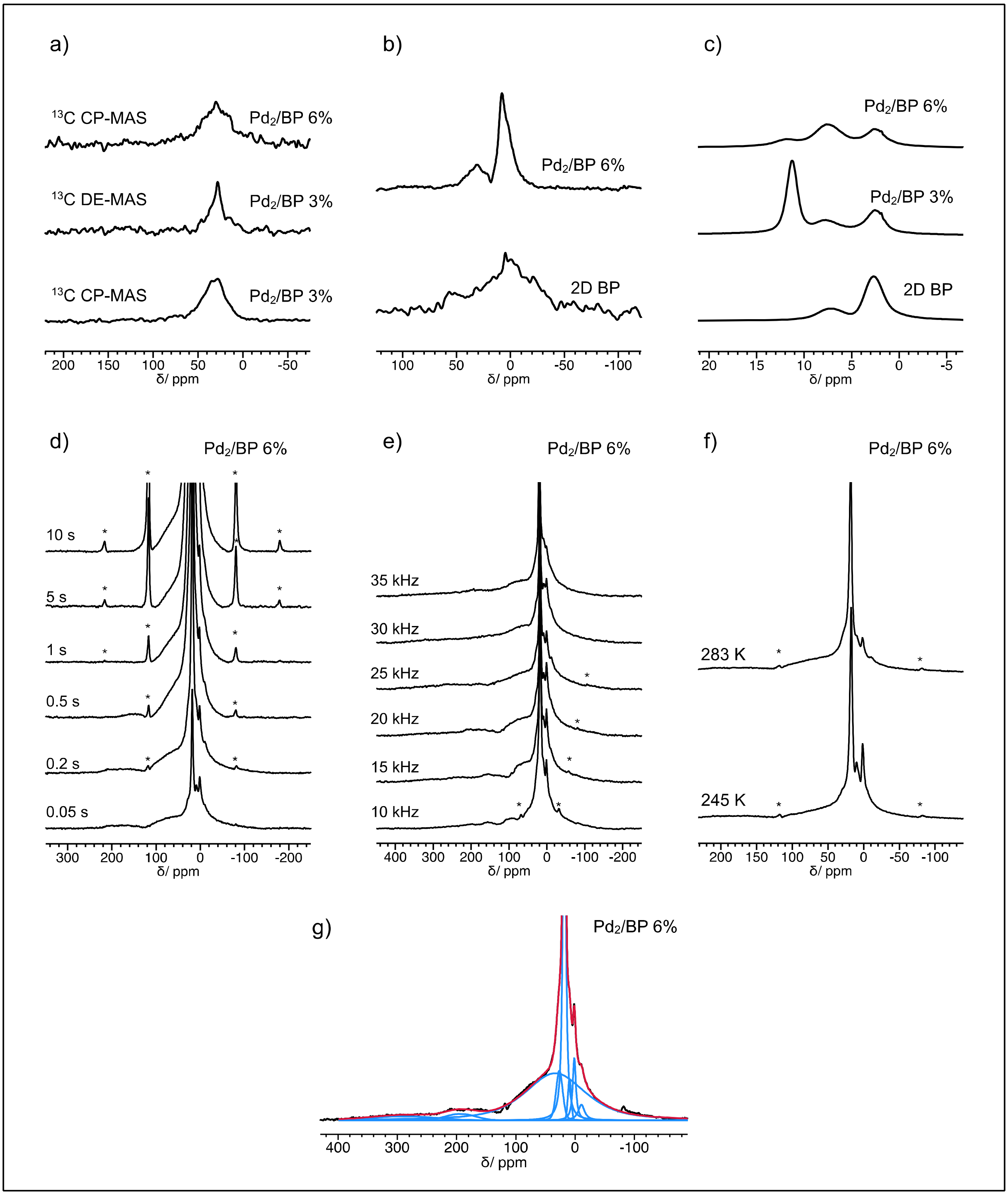


**Figure S8.** Solid State NMR spectra of 2D BP, $Pd_2$/BP 3% prepared from **1***, and $Pd_2$/BP 6% prepared from **1**. (a) $^1H$-$^{13}C$ CP-MAS spectra of $Pd_2$/BP 3% (16600 scans) and $Pd_2$/BP 6% (69000 scans) and $^{13}C$ DE-MAS spectrum of $Pd_2$/BP 3% (400 scans). (b) $^1H$-$^{31}P$ CP-MAS spectra of 2D BP (bottom; contact time 3 ms, recycle delay 10 s, MAS frequency 3 kHz, 1800 scans) and $Pd_2$/BP 6% (top; contact time 0.5 ms, recycle delay 2 s, MAS frequency 20 kHz, 13600 scans). The broad signal observed between 20 and 70 ppm, with a maximum at about 30 ppm, arises from minor amounts of alkyl groups bonded to 2D BP. Additional signals arising from minor amounts of variously protonated $HPO_3^{2-}$ and $PO_4^{3-}$ species due to oxidative degradation of 2D BP are observed between 1 and 8 ppm. (c) $^1H$ MAS spectra of, from bottom to top, 2D BP, $Pd_2$/BP 3%, and $Pd_2$/BP 6%. The intense peak at 11.3 ppm can be ascribed to P-OH hydrogen nuclei, while those at 7.7 and 2.4 ppm

arise from probehead background and alkyl species, respectively. (d) $^{31}P$ DE-MAS spectra recorded on $Pd_2$/BP 6% using the indicated recycle delay values and a MAS frequency of 20 kHz, and accumulating 1600 scans. (e) $^{31}P$ DE-MAS spectra recorded on $Pd_2$/BP 6% with 0.05 s recycle delay at the indicated MAS frequency values, and accumulating 3600 scans. (f) $^{31}P$ DE-MAS spectra of $Pd_2$/BP 6% recorded at 245 (bottom) and 283 K (top) with a recycle delay of 0.05 s and a MAS frequency of 20 kHz, and accumulating 3600 scans. Asterisks indicate spinning sidebands. (g) Calculated (red, with single peaks in blue) and experimental (black) $^{31}P$ DE-MAS selective spectrum of $Pd_2$/BP 6% (recycle delay 0.2 s, MAS frequency 20 kHz); the peaks at chemical shifts larger than 26 ppm have been used only for phenomenologically describing the composite signal of $^{31}P$ nuclei bonded to $^{105}Pd$ ones.

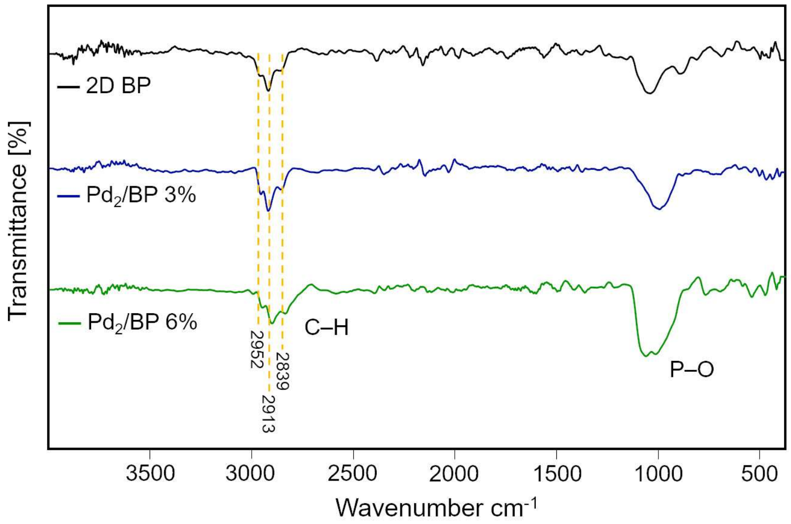


**Figure S9.** Processed ATR-FTIR spectra of $Pd_2$/BP derivatives and pristine 2D BP.

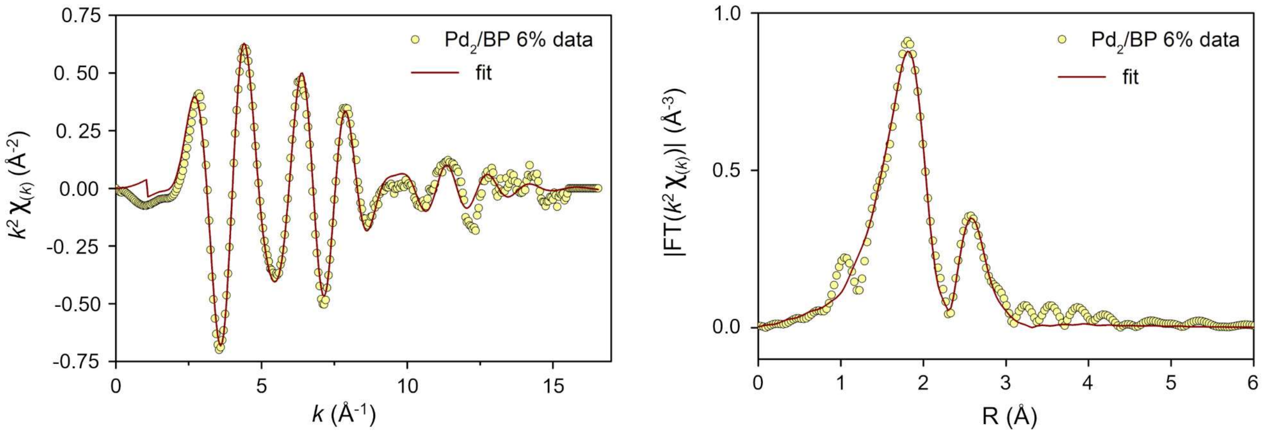


**Figure S10.** EXAFS $k^2$-weighed spectrum of $Pd_2$/BP 6% (left) and corresponding Fourier transform (right).

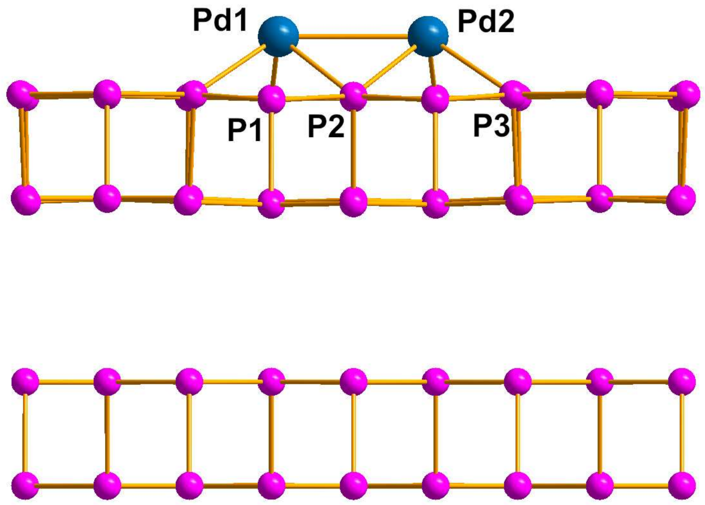


| *vector* | *length (Å)* |
|---|---|
| Pd1−Pd2 | 3.05 |
| Pd1−P1 | 2.41 |
| Pd1−P2 | 2.50 |
| Pd2−P3 | 2.32 |

**Figure S11.** Optimized geometry of the $Pd_2$ unit adsorbed on a bilayer BP surface. The adsorption of a dinuclear palladium unit on top of the BP surface has been taken into account and investigated with *ab initio* modelling. The optimized structure features a Pd–Pd distance of 3.05 Å, together with the coordination of each Pd center with three phosphorus atoms. However, this structure has been discarded for both an unfavourable structural arrangement and for being less stable than the model in Figure 5 by +37.0 kcal $mol^{-1}$.

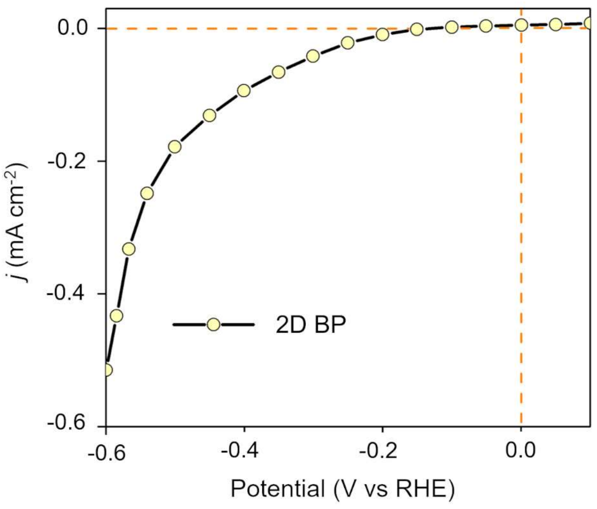


**Figure S12.** LSV activity of pristine 2D BP. Scan rate 1 $mVs^{-1}$, 1600 rpm RDE rotations.

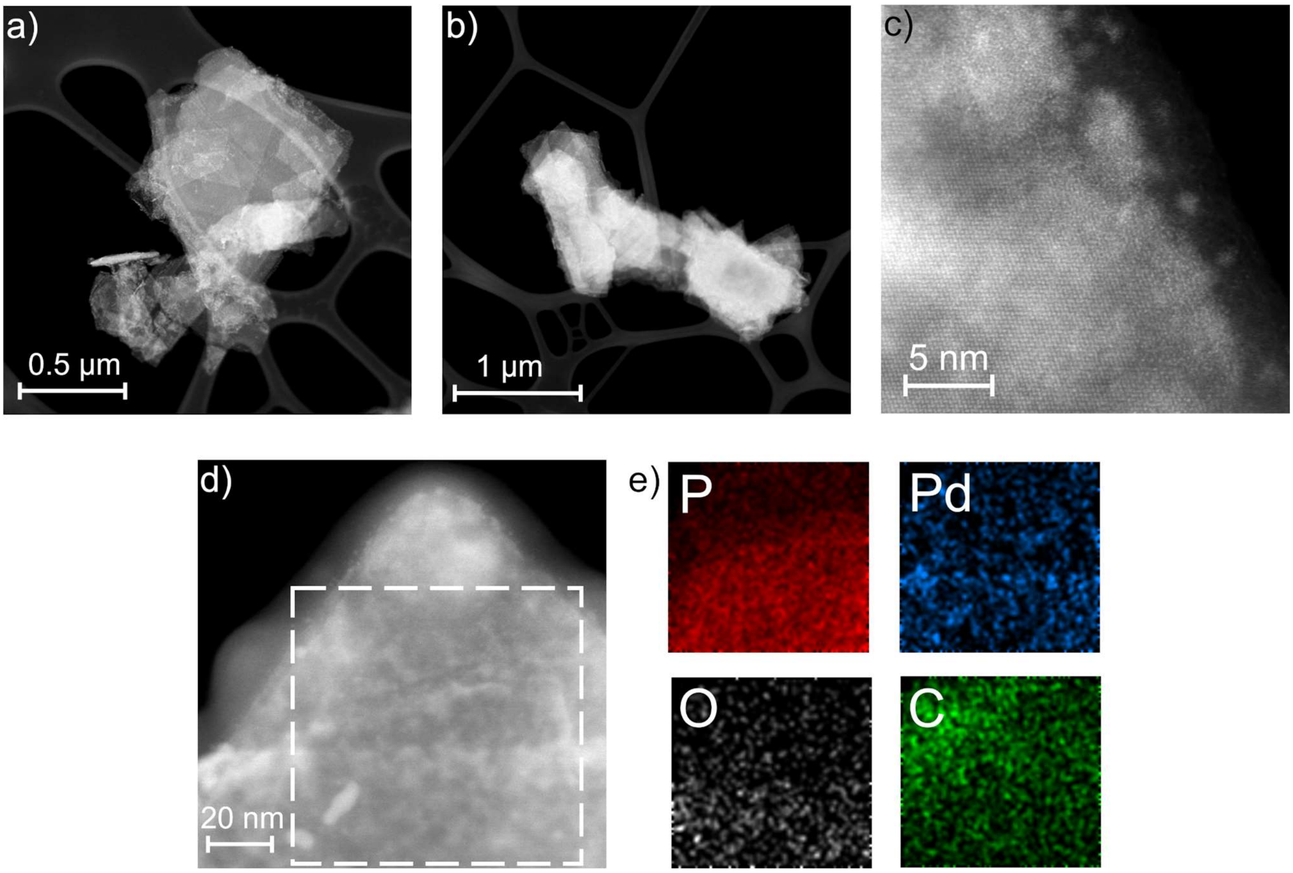


**Figure S13.** a-d) HAADF-STEM imaging of $Pd_2$/BP 3% flakes (exhaust catalyst from HER) dropcasted on a carbon grid. e) EDS elemental mapping of the region highlighted in (d).

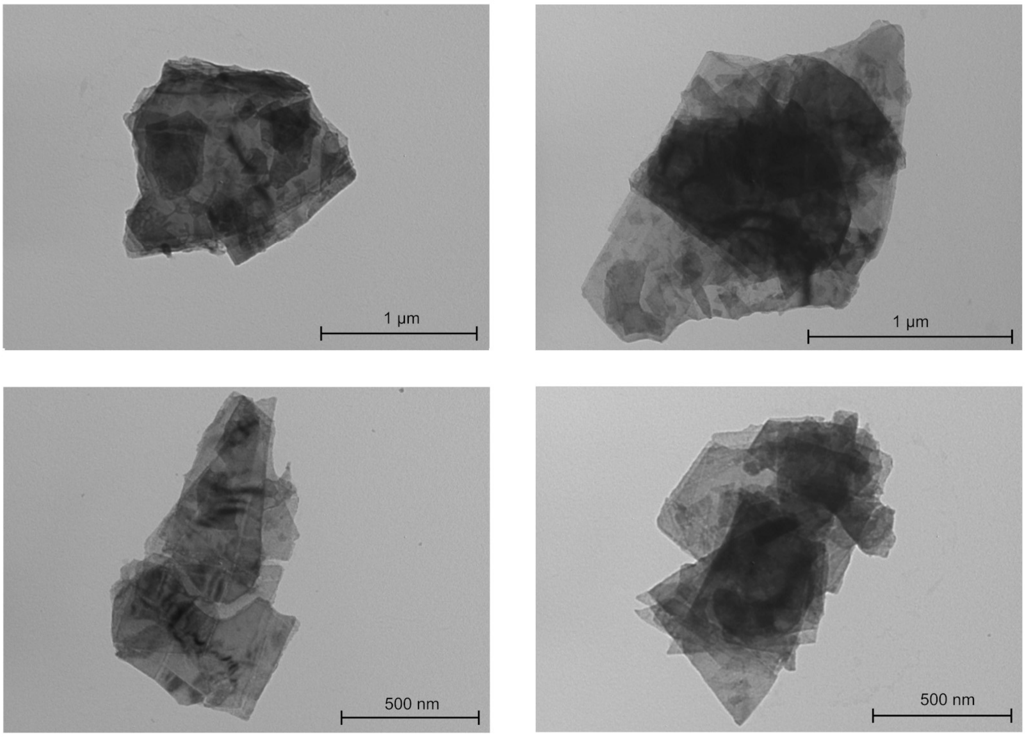


**Figure S14.** TEM characterization of the exhaust $Pd_2$/BP 3% catalyst recovered from HER measurements.

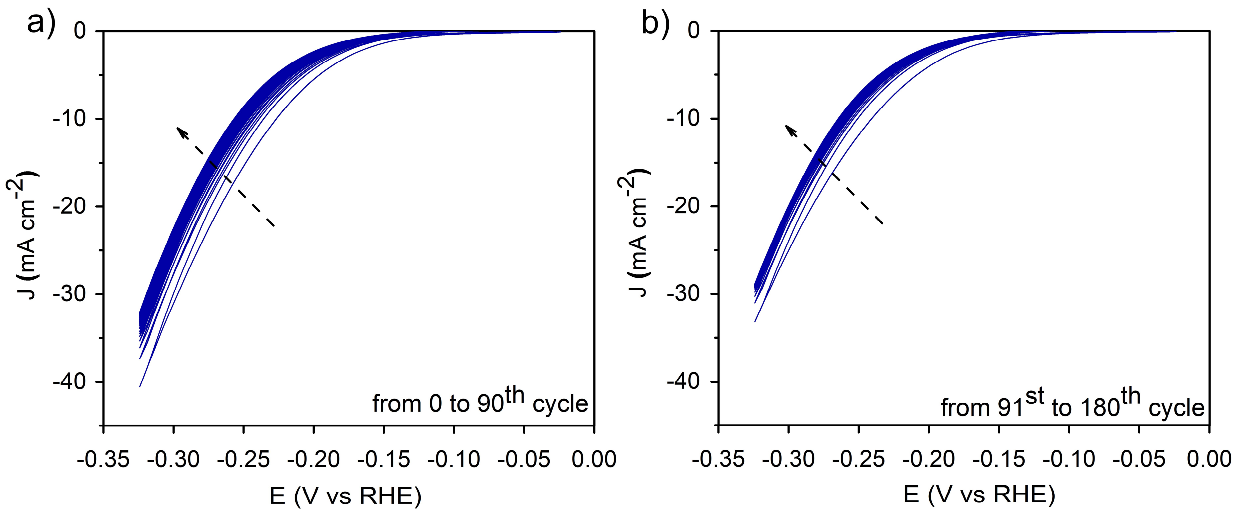


**Figure S15.** Stress test of $Pd_2$/BP 6%. a) first batch of 90 CVs. b) second batch of 90 CVs acquired after recovering and storing the working electrode for three hours under air. The tests were run at 20 mV $s^{-1}$ scan rate.

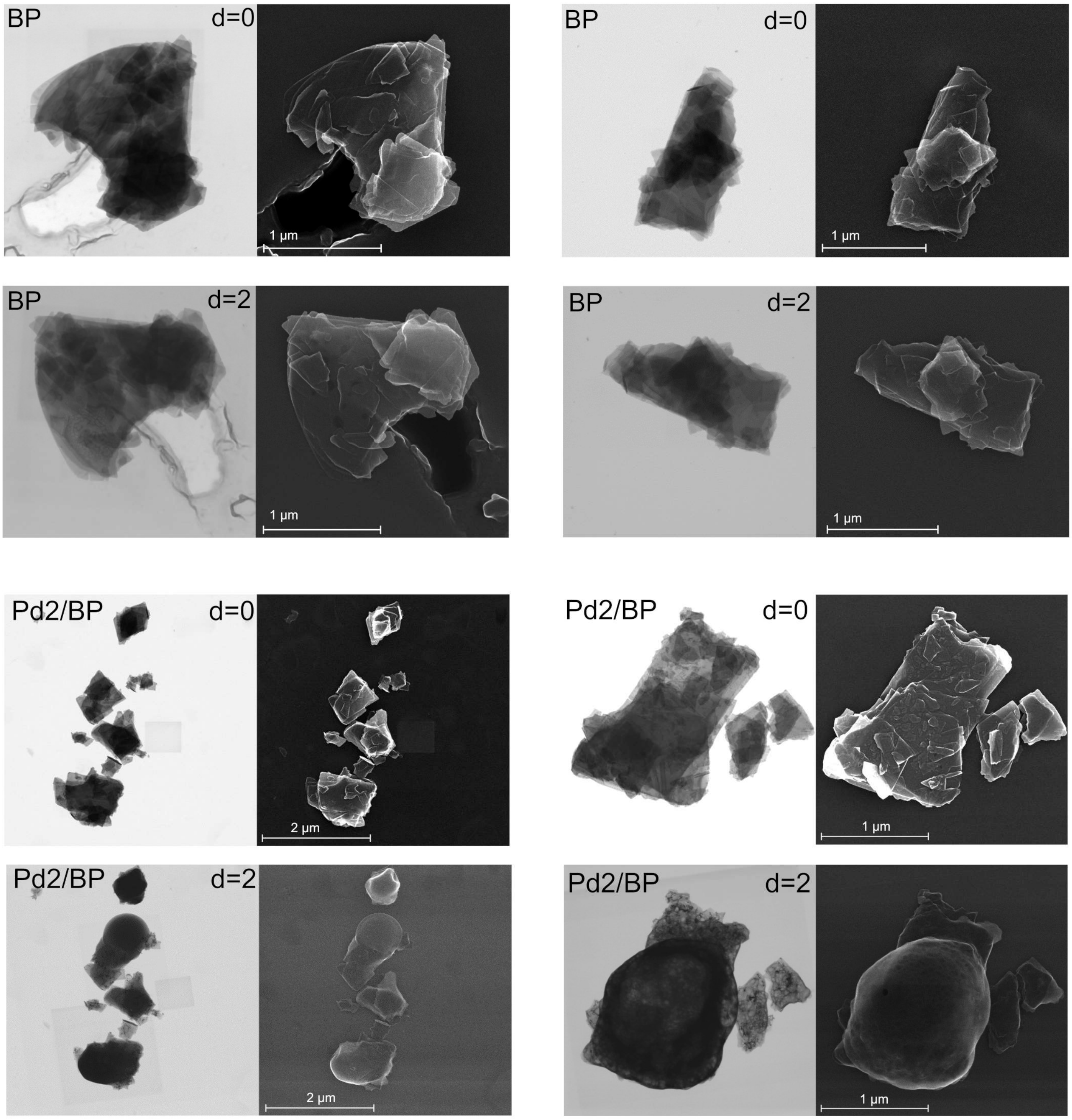


**Figure S16.** STEM and SEM analysis of $Pd_2$/BP 6% and pristine BP upon 0 and 2 days of air exposure. Whereas the morphology of BP is only slightly affected, after 2 days major alterations occur to $Pd_2$/BP flakes, looking mostly degraded and covered with large water blobs. Notably, upon degradation of the BP lattice in $Pd_2$/BP 6% metal aggregation takes place, giving rise to several clusters of particles noticeable in the STEM micrographs (presumably made of Pd or PdO).

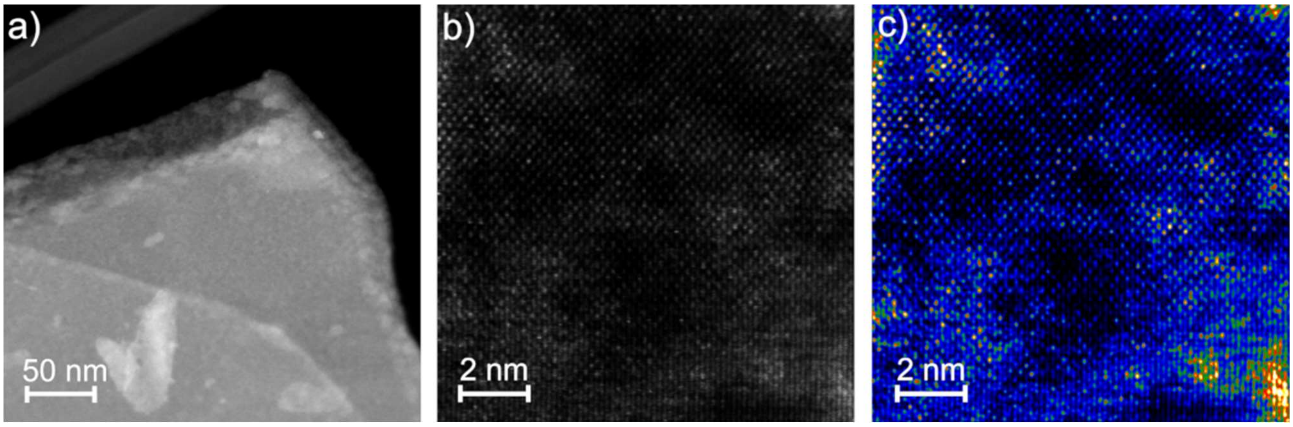


**Figure S17.** a) Detail of a flake edge. b) High-resolution Z-contrast imaging. c) False-colours display of the micrograph in (b) (warmer colours correspond to higher Z).

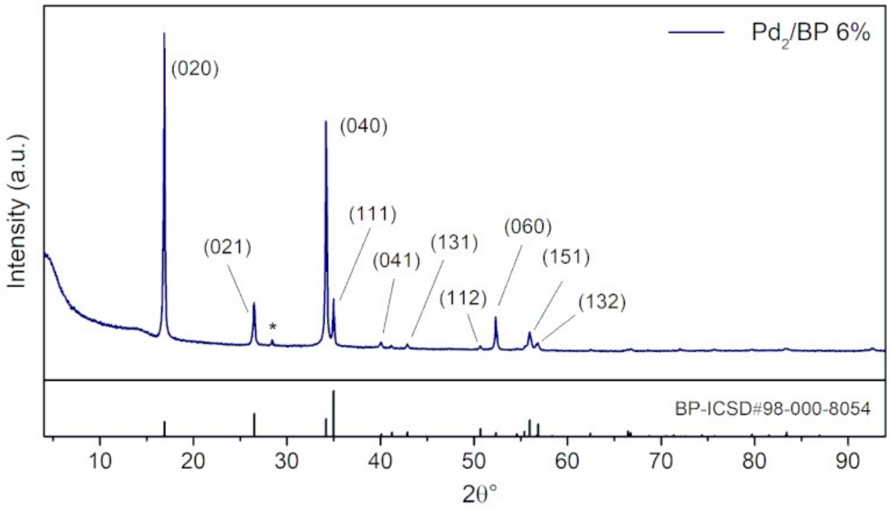


**Figure S18.** XRD spectrum of $Pd_2$/BP 6%. The reference pattern of orthorhombic BP is shown at the bottom. The peak marked with an asterisk is due to the sample-holder.

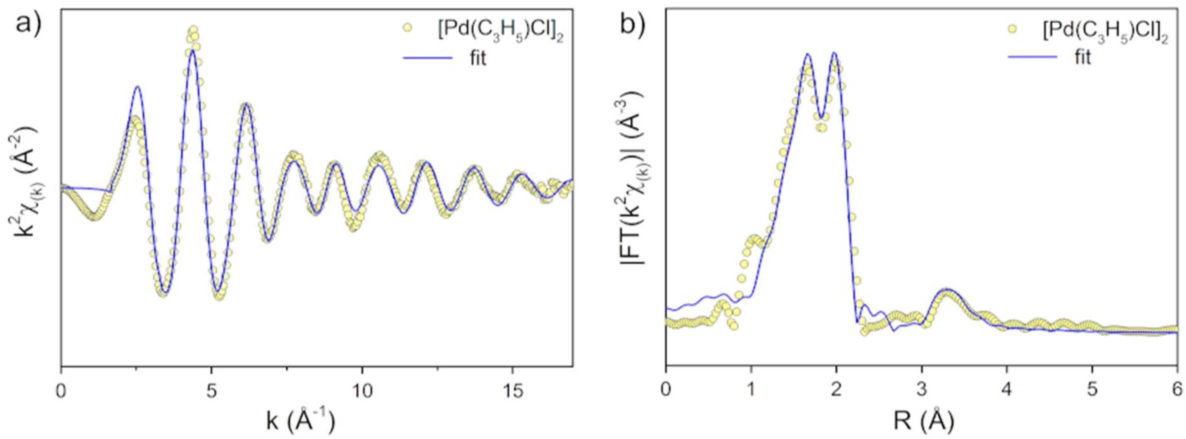


**Figure S19.** EXAFS spectrum of **1** (a) and corresponding Fourier transform (b).

# 2. Synthesis and Catalysis

**2.1 General Methods and materials.** All the syntheses and manipulations involving 2D BP were performed under inert atmosphere using Schlenk line techniques. Dichloromethane (DCM) was distilled from $CaH_2$, stored above molecular sieves 3 Å and degassed prior to use. Red phosphorus (> 99.99%), phenylacetylene, 1-octene and allyl chloride were purchased from Sigma Aldrich. $PdCl_2$ was purchased from Strem Chemicals. 1-$^{13}$C-allyl alcohol (99%$_{at}$ $^{13}$C) was purchased from Sigma Aldrich (100 mg batch).

**2.2 BP synthesis and exfoliation.** Bulk BP crystals were prepared following the literature method reported by T. Nilges *et al.*,[S3] which allows BP to be prepared via chemical vapour transport (CVT), starting from red phosphorus as P-source and Sn/$SnI_4$ as mineralizing agents. Liquid phase exfoliation (LPE) of BP was carried out in dry DMSO ($H_2O$ < 30 ppm), working on a 5 mg batch scale, following a literature method with minor modifications.[S4] BP crystals and deoxygenated DMSO (BP/DMSO = 1 mg/mL) were transferred inside a borosilicate glass tube (length = 300 mm, outer diameter = 15 mm; inner diameter = 11.3 mm), then a controlled amount of deoxygenated water was added and the ampoule was sealed under inert atmosphere. The ampoule was dipped inside an ultrasonic bath (37 kHz, 80% power) and sonicated over 6 days at 30°C. After this time, a dark brownish dispersion was obtained. As probed via AFM and TEM analysis, the resulting flakes have lateral dimensions within the range 300-900 nm and average thickness below 30 nm. Prior to functionalization, the exfoliated material was washed to eliminate DMSO. In detail, the suspension resulting from sonochemical exfoliation (5 mg/5 $mL_{DMSO}$) was centrifuged at 9500 rpm for 30 minutes to isolate 2D BP as a solid residue (the supernatant was discarded). The solid was resuspended in deaerated ethanol using ultrasounds (5 min), before being recovered after centrifugation. This washing cycle was repeated 4 times in total, using acetone in the 4$^{th}$ step. The solid was dried under vacuum for 24 h, before an additional final washing with distilled and deaerated DCM was performed.

**2.3 Synthesis of $[Pd(C_3H_5)Cl]_2$ (1).** Complex **1** was prepared according to a literature method[S5] and carefully purified by filtration of the first obtained **1** crop dissolved in anhydrous DCM over neutral $Al_2O_3$. Upon reduction of the filtered solution to a small volume and addition of dry *n*-pentane, pure **1** was obtained as a yellow crystalline powder (yield 81%). *$^1$H NMR (400 MHz, $CD_2Cl_2$, 298K):* δ = 3.05 (d, $^2J$ = 12.16 Hz, 2H), 4.10 (d, $^3J$ = 6.74 Hz, 2H), 5.50 ppm (m, 1H).

**2.4 Synthesis of $[Pd(1\text{-}^{13}C\text{-}C_3H_5)Cl]_2$ (1*).** The synthesis was carried out in two steps: first, the labelled allyl alcohol-1-$^{13}$C (AA*) was converted in the $^{13}$C-enriched allyl chloride-1-$^{13}$C (AC*) and, second, the $^{13}$C-labelled dimer **1*** was synthesised from AC* (scale-down of the procedure 2.3). Since the overall purchased AA* amounted to 100 mg (~117 μL), a straightforward synthetic protocol to convert AA* in AC* avoiding any intermediate purification step (*i.e.* distillation) was necessary, which imposed to revisit

and improve a patented procedure.[S6] The whole protocol was set up and checked using non-labelled AA before repeating the synthesis with AA*.

*Step 1)* 11 mg of $PdCl_2$ (0.062 mmol, used as catalyst) were added to a short NMR tube, used as reactor, followed by 0.56 mL of concentrated HCl 37%. Once the solid was dissolved, 100 mg of AA* (1.72 mmol, $[AA^*]^{1/2}/[HCl] = 0.19$) were added, the tube was sealed, a customized refrigerator was mounted on top (see Figure S20) and the solution was heated to 80°C for 1 h. During this time, a colourless organic phase (AC*) was formed which separated above the reddish acidic phase.

*Step 2)* A glass vial equipped with a magnetic stirring bar was charged with 90 mg of $PdCl_2$ (0.51 mmol, 1 eq) and 68 mg of NaCl (2.3 eq), followed by 410 µL of distilled water. The mixture was stirred until the palladium salt was dissolved forming soluble $Na_2PdCl_4$. Then, the acidic red phase at the bottom of the NMR tube was removed by syringe and discarded. 1.5 mL of MeOH were added to rinse the tube collecting AC* and then transferred to the vial containing $Na_2PdCl_4$. Further 1.0 mL of MeOH was added, then CO was slowly bubbled in the solution under stirring over 1 h (see Figure S20). A colourless precipitate formed (NaCl) while the solution turned yellow. The suspension was then poured into water (13 mL) and extracted with DCM (3 x 5 mL). The organic phases were collected altogether and washed with water (2 x 6 mL), then left 1 h over anhydrous $MgSO_4$. The organic phase was concentrated to *ca.* 10 mL under a nitrogen stream and filtered over $Al_2O_3$ (via a Pasteur pipette packed with ~ 1 cm of $Al_2O_3$). The filtrate was reduced to a small volume and *n*-pentane was added to precipitate **1*** as a yellow crystalline powder (71 mg, 76% yield *vs* initial $PdCl_2$).

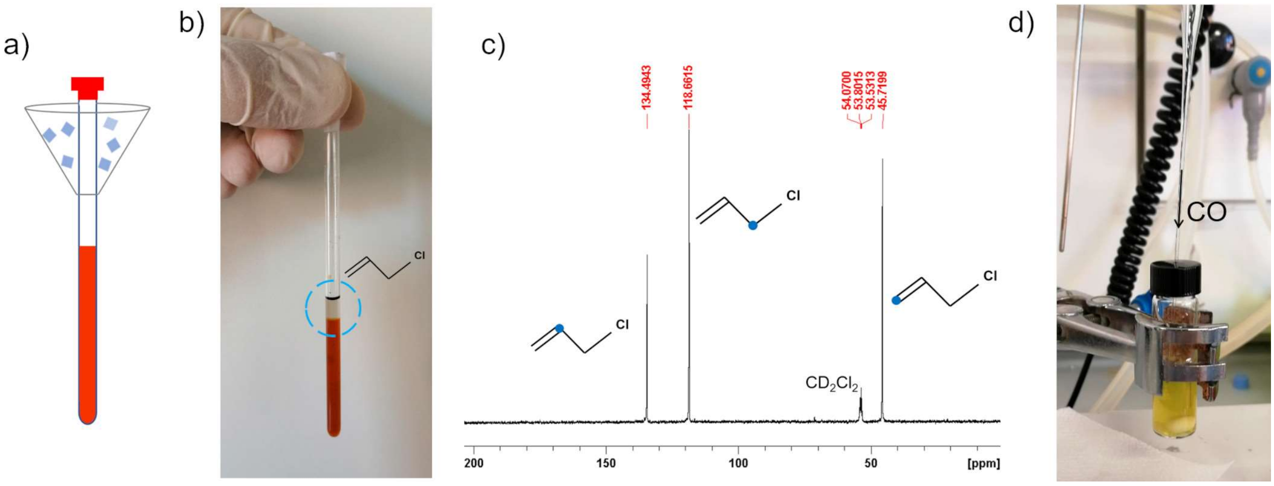


**Figure S20.** a) Schematic representation of the NMR tube-reactor with an ice-refrigerator (the drilled bottom of a PP centrifuge tube) mounted on top. b) AC* layer formed on top of the acidic aqueous phase after 1 h at 80°C. c) $^{13}C$ NMR spectrum of the organic layer (corresponding to a trial with unlabelled AC) in $CD_2Cl_2$, showing the presence of the only AC. d) Formation of the yellow complex **1*** upon CO bubbling through the solution containing $Na_2PdCl_4$ and AC*.

*$^{1}H$ NMR ($CD_2Cl_2$, 400 MHz, 298 K).* The $^{1}H$ NMR spectrum is reported in Figure S21a. $^{13}C$ labelling of a terminal atom in the allylic moiety turns the allylic $A_2E_2M$ spin system of **1** into a second order AA'EE'MX system (see the labelling scheme depicted in the Figure). A simulation of the experimental spectrum was carried out with SpinWorks 4, with the following *J* (Hz) values:

| | | |
|---|---|---|
| J(A,E) = 0.60 | J(E,M) = 6.74 | J(M,E') = 6.74 |
| J(A,M) = 12.16 | J(E,A') = 0.50 | J(M,X) = 3.60 |
| J(A,A') = 1.00 | J(E,E') = 2.15 | J(A',E') = 0.60 |
| J(A,E') = 0.50 | J(E,X) = 161.00 | J(A',X) = 5.80 |
| J(A,X) = 160.00 | J(M,A') = 12.16 | J(E',X) = 9.35 |

*$^{13}C\{^{1}H\}$ NMR ($CD_2Cl_2$, 400 MHz, 298K).* The $^{13}C$ NMR spectrum is shown in Figure S21b. The intense singlet at $\delta = 62.67$ ppm corresponds to the labelled terminal carbon, the small doublet ($^{1}J_{CC} = 43.6$ Hz) corresponds to the central carbon spin-coupled to the labelled one.

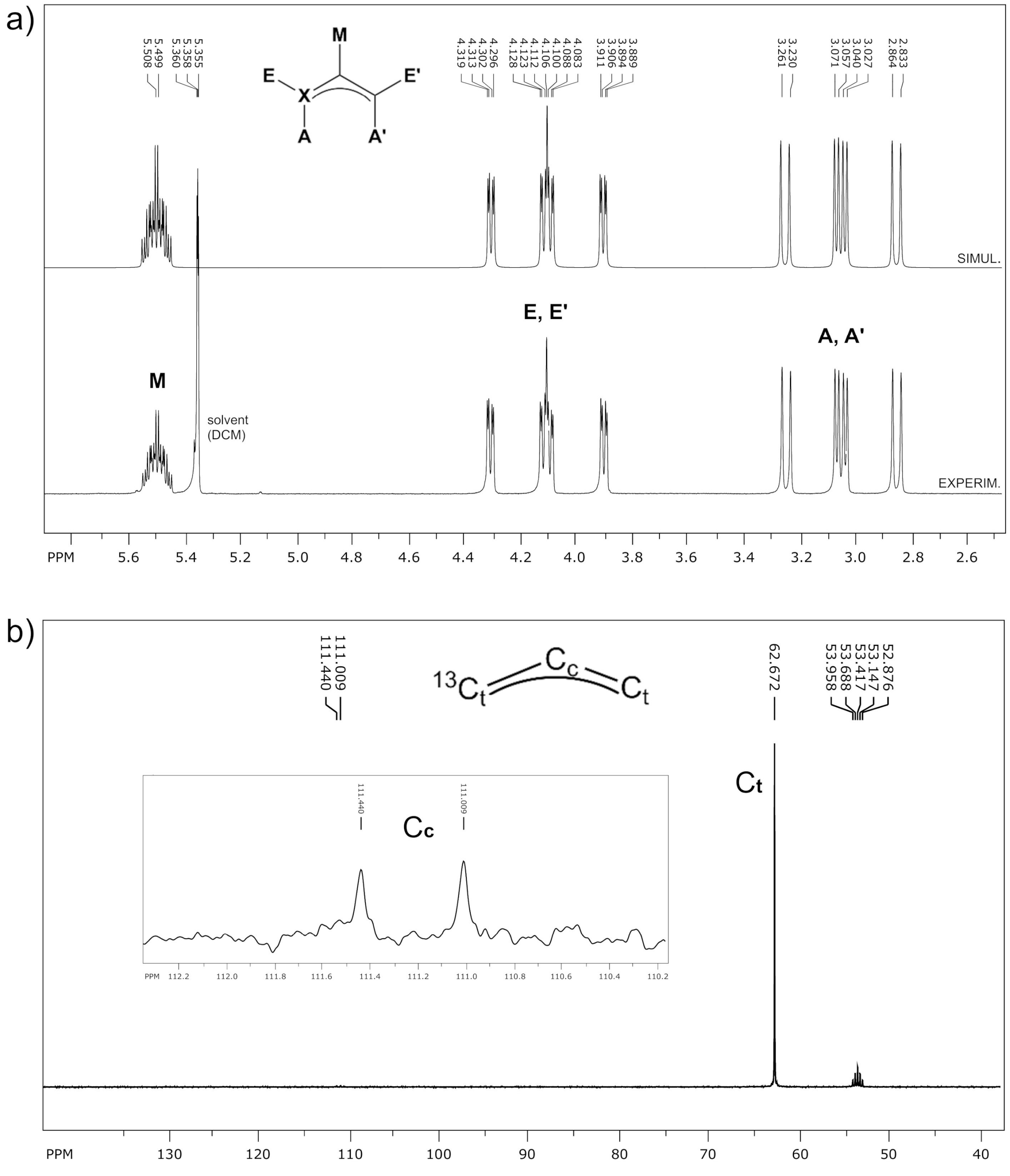


**Figure S21.** a) $^1$H and (b) $^{13}C\{^1H\}$ NMR spectra of **1*** ($CD_2Cl_2$, 400 MHz, 298 K).

**2.5 2D BP functionalization with 1.** In a typical procedure, 5 mg (0.161 mmol) of 2D BP were suspended in 8 mL of DCM with ultrasonic treatment and the resulting suspension was added under inert atmosphere to a Schlenk flask charged with 6 mg of **1** (0.033 mmol, P/Pd molar ratio = 5) and a magnetic stir bar. The mixture was stirred in the dark for 18 h at RT. After this time, the suspension was transferred under inert

atmosphere in a centrifuge tube and the solid material ($Pd_2$/BP) was collected by centrifugation (9500 rpm x 30 min). Then it was redispersed in fresh DCM (8 mL) with the aid of a spatula, and isolated by centrifugation. This last washing cycle was repeated 3 times in total. The solid was then dried in vacuum and stored under inert atmosphere. ICP-AES analysis revealed a Pd/P molar ratio of 3.3 % (material named $Pd_2$/BP 3%). Carrying out the reaction under reflux (~ 40 °C) gave a Pd content of 6.2 % (material named $Pd_2$/BP 6%).

**2.6 2D BP functionalization with 1*.** Functionalization was carried out as described in 2.5 working at RT with **1***, scaling-up the synthesis to 20 mg of 2D BP. Two identical functionalizations were run in parallel and the resulting $Pd_2$/BP batches were collected for a total of 40 mg of $Pd_2$/BP (mass referred to the BP content).

**2.7 Hydrogenation of phenylacetylene.** A glass vial equipped with a magnetic stirring bar was charged with a suspension of $Pd_2$/BP 3% (3 mg in 4 mL). Phenylacetylene was added (32 μL, 0.3 mmol, S/C=100). The vial was transferred inside a Parr autoclave. The autoclave was sealed, purged four times with $H_2$, pressurized to 10 bar and stirred at RT for 1 h. After this time, the volatiles were vented out and the autoclave was opened. The obtained suspension was centrifuged, and the liquid phase was collected for GC-MS analysis. Conversion = 0%.

**2.8 Hydrogenation of 1-octene.** *Test 1.* A catalytic trial was carried out as described above using 2 mg of $Pd_2$/BP 3% and 30 μL of 1-octene (0.19 mmol, S/C=100). The autoclave was pressurized with 10 bar of $H_2$ and kept stirring at RT for 2 h. Conversion = 0%. *Test 2.* A second test was carried out on 1 mg of $Pd_2$/BP 6% suspended in 4 mL of DCM and 30 μL of 1-octene (0.19 mmol, S/C=100). The autoclave was pressurized with 5 bar of $H_2$ and kept stirring at room temperature for 17 h. Conversion = 0%.

**2.9 Electrocatalytic measurements.** All the glassware was cleaned with an $H_2O_2$/$H_2SO_4$ solution (1/3 $H_2O_2$ 30% + 2/3 $H_2SO_4$ 98%) overnight and rinsed several times with Milli-Q water prior to use. The working electrode, a RDE glassy carbon disk (0.1963 $cm^2$) embedded in a PTFE jacket (PINE™) was cleaned by stirring overnight in a 0.05 μm alumina aqueous suspension. After this treatment the electrode was washed in sequence in acetone, 2-propanol and Milli-Q water. All the aqueous solutions were prepared with Milli-Q water (18.5 MΩ*cm at 25°C) provided with a Millipore Milli-Q3 apparatus (Nihon Millipore Ltd.). Chemicals were used as purchased from Sigma-Aldrich/Merk. Polarization curves and chronopotentiometries performed in aqueous solutions were acquired in a standard pyrex three-electrode cell (Princeton Applied Research). The reference electrode was a commercial Ag/AgCl/$KCl_{(sat)}$ (Princeton Applied Research) and the counter electrode was a gold gauze enclosed in a glass tube with porous bottom. The RDE working electrode WE was coated by drop casting 34 μL of the catalyst ink by means of a micropipette. The catalyst layer was then dried under pure nitrogen flow and covered by a thin Nafion

film (0.5% wt in 2-propanol) acting as binder to stick the catalyst onto the glassy carbon disk during the RDE rotation at 1600 rpm. All electrochemical studies were carried out at room temperature (20-25°C) using a Parstat 2273 potentiostat–galvanostat (Princeton Applied Research) equipped with a Model 616 Rotating Disk Electrode (PAR/Ametek). All the potentials were reported versus the Reference Hydrogen Electrode RHE.

*Polarization experiments.* Polarization (aka linear sweep voltammetry, LSV) experiments were performed in a 0.5 M $H_2SO_4$ aqueous solution (pH 0.3) saturated with hydrogen (30 minutes of pure hydrogen bubbling) with 1 mV $s^{-1}$ scan rate, rotating the WE at 1600 rpm. The hydrogen evolution reaction was evaluated performing the scans between 0.1 and -0.4 V *vs* RHE without compensating the resistance. The LSV of $Pd_2$/BP was repeated several times with different depositions batches, showing a strong reproducibility of the measurements, except at high cathodic potentials where the formation of hydrogen bubbles interrupts the current circulation in the cell. For this reason, it was not possible to push up the LSV at potentials more negative than -0.4 V *vs* RHE.

*Cyclic Voltammetry.* Cyclic Voltammetries (CVs) were performed in a 0.5 M $H_2SO_4$ aqueous solution purged for 30 minutes with pure hydrogen bubbling. Voltammetries were acquired between 0 and -0.325 V *vs* RHE with 20 mV $s^{-1}$ scan rate, rotating the WE at 2600 rpm. Any resistance compensation was operated.

*Chronopotentiometric experiments.* Chronopotentiometric measurements (galvanostatic experiments) were carried out in a 0.5 M $H_2SO_4$ aqueous solution (pH 0.3) saturated with hydrogen. The experiments were performed at the constant current load of -1 mA (-5 mA $cm^{-2}$) rotating the working electrode at 1600 rpm for 3600 seconds.

*Ink preparation.* The ink was prepared in a glass vial suspending the catalyst (2D BP or $Pd_2$/BP) in DCM with ultrasounds for 1 min in cold water to slow down DCM evaporation during ink deposition. The suspensions concentration spans between 3.2 to 3.5 mg $mL^{-1}$ and before each deposition the inks were re-suspended with ultrasounds for 1 min.

# 3. Characterization of the material

**3.1 Inductively coupled plasma-Atomic emission spectroscopy (ICP-AES).** ICP-AES measurements were performed with an Agilent 7700 Series spectrometer at the Chemistry Department, University of Florence (Italy). Samples followed a microwave-assisted digestion in nitric acid for trace analysis. Then, different dilutions of each sample with water for trace analysis were prepared, in order to obtain concentrations in the sensitivity range of the instrument for the elements under investigation (namely Pd and P). Standards at different concentrations were also prepared and measured contextually to sample measurements, in order to obtain a calibration curve for each element under investigation.

**3.2 TEM microscopy.** Transmission Electron Microscopy studies were carried out at Ce.ME CNR (Sesto Fiorentino, Italy) using a Philips CM12 electron microscope operating at an accelerating voltage of 80 kV. Few drops of $Pd_2$/BP suspended in DCM were placed on the TEM copper/carbon grid, dried under a stream of nitrogen, and measured.

**3.3 SEM microscopy.** Scanning Electron Microscopy experiments were carried out at Ce.ME CNR (Sesto Fiorentino, Italy) using a Dual Beam, TESCAN GAIA3 FIB/SEM ultrahigh resolution field emission microscope at 5 KeV voltage. Few drops of $Pd_2$/BP suspended in DCM were placed on the TEM copper/carbon grid, dried under a stream of nitrogen, and measured.**3.4 HAADF-STEM, EDS and EELS spectroscopy.** Atomic-resolution characterization by STEM was performed at CNR- IMM (Catania, Italy) through a probe aberration-corrected JEOL ARM200CF, equipped with a Ceos hexapole-type Cs corrector, named CESCOR, and operated at a primary beam energy of 60 keV. The electron gun is a cold-field emission gun with an energy spread of 0.3 eV. The probe size was 1.1 Å at 60 kV. Micrographs were acquired in Z-contrast mode (High-Angle Annular Dark Field, HAADF).

A Centurio Energy Dispersive Spectrometer (EDS) equipped with a 100 $mm^2$ Silicon Drift Detector was used for the EDS acquisitions.

A GIF Quantum ER as Electron Energy Loss Spectrometer (EELS) was used for EELS measurements. Both low- and core-loss EELS spectra were acquired with the DualEELS capability through Gatan Digital Micrograph software, which allows the accurate energy calibration of EELS spectra, thanks to the simultaneous alignment of the zero-loss peak position for every single acquisition which removes any artefact coming from energy shifts. The use of Fourier logarithmic deconvolution on a full spectrum obtained by splicing together low- and core-loss EELS allows removing thickness-related plural scattering. All the STEM-EELS and STEM-EDS measurements were performed simultaneously by using the Gatan spectrum imaging (SI) tool.

**3.5 Powder X-ray Diffarction (XRD).** Data were collected with an X'Pert PRO diffractometer, operating in Bragg-Brentano parafocusing geometry with Cu-Kα radiation (λ = 1.5418) at 40 kV and 30mA. Samples were prepared slowly drop-casting the material suspended in DCM, while directing a nitrogen stream onto the sample holder to speed up solvent evaporation. The process was continued until a uniform film of the material had formed. Data acquisition was carried out under air exposure.

**3.6 Raman scattering.** Raman measurements were carried out at CNR-IFAC (Florence, Italy) using a micro-Horiba Xplora system coupled to a 532 nm wavelength laser. The backscattered light was collected by a 100× microscope objective with 0.9 NA, which generates a ~1 μm large laser beam waist. Integration times of 10 s, laser power values in the 1-2 mW range and a grating of 1200 $cm^{-1}$ were employed. The samples were prepared by dropcasting a suspension of 2D BP or $Pd_2$/BP in DCM on a Si/$SiO_2$ wafer. After one minute of exposure, the wafers were rinsed with DCM and dried under a stream of nitrogen for 15 min. The Raman spectra displayed in Figure 3 were obtained averaging the data recorded from 15 individual flakes randomly chosen from each dropcasted sample. A second and larger statistical analysis was also carried out using 60 spectra for both $Pd_2$/BP and pristine BP. The latter were collected choosing 60 random points from larger (*ca.* 20 μm in diameter) multi-flake aggregates on the silicon wafer. The results of this analysis are reported in Figure S2.

**3.7 ATR-FTIR.** Attenuated total reflectance (ATR) FT-IR spectra were recorded under air with a Perkin-Elmer Two Spectrometer, equipped with an ATR unit with diamond crystal. Spectra acquisition was carried out with a resolution of 4 $cm^{-1}$ using 64 scans.

**3.8 X-ray Photoelectron Spectroscopy (XPS).** X-ray Photoelectron Spectroscopy (XPS) measurements were performed at the Chemistry Department, University of Florence (Italy) in an ultra-high vacuum ($10^{-9}$ mbar) system equipped with a VSW HAC 5000 hemispherical electron energy analyzer and a non-monochromatized Mg-Kα X-ray source (1253.6 eV). The source power used was 100 W (10 kV×10 mA) and the spectra were acquired in the constant-pass-energy mode at $E_{pas}$ = 44 eV. The overall energy resolution was 1.2 eV as a full-width at half maximum (FWHM) for the Ag $3d_{5/2}$ line of a pure silver reference. The recorded spectra were fitted using XPS Peak 4.1 software employing Gauss-Lorentz curves after subtraction of a Shirley-type background. The samples were dropcasted above the sample holder from a suspension in DCM, dried under a stream of nitrogen and introduced in the UHV system via a loadlock under inert gas ($N_2$) flux, in order to minimize the exposure to air contaminants and kept in the introduction chamber for at least 12 hours before the measurements.

**3.9 Solid State NMR Spectroscopy.** Solid State NMR spectra were recorded on a Bruker Avance Neo spectrometer working at Larmor frequencies of $^{1}H$, $^{13}C$ and $^{31}P$ nuclei of 500.13, 125.77, 202.46 MHz, respectively, using a triple-resonance Cross Polarization-Magic Angle Spinning (CP-MAS) probehead

accommodating rotors with an external diameter of 2.5 mm. $^{31}P$ MAS spectra were recorded both using a Direct Excitation (DE) pulse sequence and a ($^{1}H$-$^{31}P$) CP sequence under High Power Decoupling (HPD) from $^{1}H$ nuclei. $^{31}P$ DE-MAS spectra were performed at different MAS frequencies (from 5 to 35 kHz) and/or using different recycle delay values (from 0.05 s to 200 s) between consecutive transients. A different number of transients were accumulated depending on the experimental conditions used in recording the spectrum. $^{13}C$ MAS spectra were recorded using both ($^{1}H$-$^{13}C$) CP (with a contact time of 1 ms and a recycle delay of 3 s) and DE pulse sequences (with a recycle delay of 5 s), under HPD from $^{1}H$ nuclei. The $^{1}H$ MAS spectra were recorded at a MAS frequency of 20 kHz, accumulating 16 transients with a recycle delay of 2 s. Spectra were recorded at 283 K if not otherwise specified.

**3.10 X-ray Absorption Spectroscopy (XAS).** Measurements at the Pd-K edge (E = 24350 eV) were performed on the beamline SAMBA[S7] at synchrotron SOLEIL (Saint Aubin, France). The beamline is equipped with a sagittal focusing monochromator with a pair of Si(220) crystals and two Pd coated bendable cylindrical mirrors. Mirrors were used to vertically collimate (1st) and focus (2nd) the beam and act as a low energy band pass filter. The beam size at sample position is of about 300x200 $\mu m^2$ (HxV). Data were collected in continuous scan mode, each spectrum taking 150 seconds and averaging about 50 spectra. The absorption coefficient was measured in fluorescence mode using a four elements ME4 Vortex silicon drift detector. Ru, Fe and Al filters were used to reduce unwanted fluorescence and scattered radiation, they were placed as close as possible to the detector window. A Pd foil placed between the second and third ionization chamber was used as an energy calibration reference, a first ionization chamber placed before the sample was used to normalize the fluorescence signal. XAS data were reduced and analyzed with the ATENA/ARTEMIS codes[S8] whereas the theoretical XAS signals were generated with the FEFF-8.4 code[S9] using muffin tin potentials with the electron densities calculated with a Self Consistent routine and the Hedin-Lundqvist approximation for the potential energy-dependent part. Structural parameters were obtained by data fits in R space with the transformation ranges in k space varying from case to case and a $k^2$ weighing factor. *Sample preparation*: all materials were manipulated under argon inside a glove box. The samples to be analyzed were diluted with h-BN, transferred inside a die and made into pellets with a press. Each pellet was fixed to the sample holder using Kapton tape (see Figure S21). During data acquisition, the sample holder was covered with a plastic bag continuously fluxed with argon to keep it under inert atmosphere for the whole measurement.

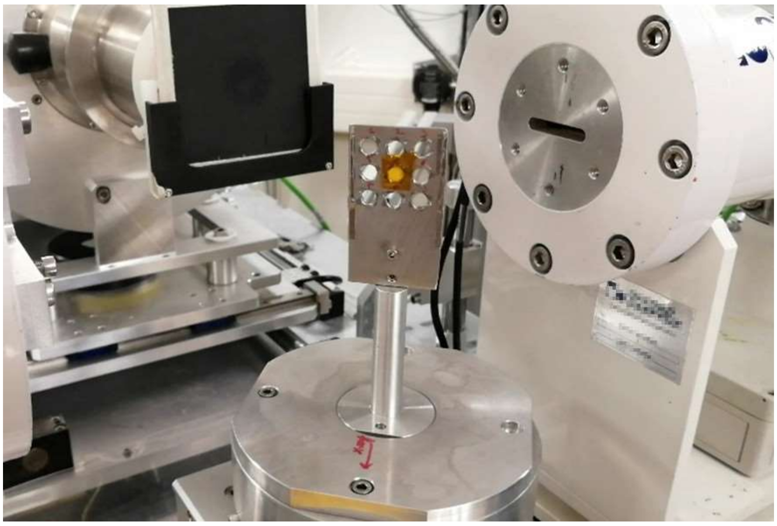

**Figure S22.** Sample holder with mounted sample, sticked between Kapton tape (orange). A plastic glove transparent to X-rays continuously fluxed with argon (not shown in the figure) was put over the sample holder during data acquisition.

Comparison between the structural parameters of **1** obtained from EXAFS fitting (Table S2) and single crystal literature data (the values are reported in the molecular structure below, CCDC 1102405):

| **Table S2** | | | |
|---|---|---|---|
| **Path** | **CN** | **r (Å)** | **$\sigma^2$ (Å$^2$)** |
| Pd–C | 1.6(3) | 2.11(1) | 0.0014(2) |
| Pd–Cl | 2.0(3) | 2.41(1) | 0.006(2) |
| Pd–Pd | 3(2) | 3.48(3) | 0.019(8) |

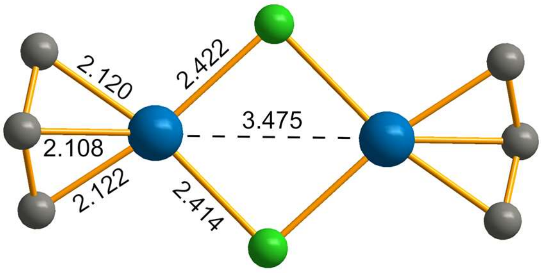


EXAFS data reproduce perfectly the interatomic distances observed by single crystal diffraction, including the non-bonding 3rd shell Pd–Pd distance. The underestimation of the Pd–C coordination number is not a peculiarity of this measurement, since it was observed earlier in molecular allylic complexes of Rh and Ir.[S10,S11]

**3.11 GC-MS analysis.** GC analyses were performed on a Shimadzu GC-2010 gas chromatograph equipped with a flame ionization detector and a 30 m (0.25 mm i.d., 0.25 mm film thickness) Varian VF-WAXms apolar capillary column. GC-MS analyses were performed on a Shimadzu QP2010SE spectrometer equipped with an identical capillary column.

**3.12 DFT calculations.** *Computational details.* The optimized geometries and energetics of the molecular model of naked and functionalized BP have been studied at B3LYP-DFT[S12],[S13] level of theory by using the CRYSTAL17 package.[S14] A *pseudo*-potential[S15] has been used for the Pd centers while the TZVP basis set has

been employed for all the atomic species. Band and Density of State (DOS) analyses have been carried out with the available routines of the CRYSTAL17 package.[S14]

***Optimized structure of the interlayer $Pd_2$/BP adduct (Figure 5 in the main text).***

CELL 10.05429319 9.45423848 90.311130

P 3.045244372984E-02 -4.064193713984E-02 5.694898928143E-01
P -4.693200976199E-01 1.069312148062E-01 4.752562966395E-01
P -4.687889633869E-01 1.971071899325E-01 -1.661502954720E+00
P 3.171462170920E-02 3.599103823838E-01 -1.652731117582E+00
P 1.990170064904E-01 1.150597504856E-01 4.346223379134E-01
P 1.968655624537E-01 2.009331510243E-01 -1.700599226747E+00
P 3.236819579978E-02 4.504234478326E-01 4.841806263413E-01
P -4.691354229881E-01 -3.956533414617E-01 4.816021762097E-01
P -4.692890655142E-01 -2.998783800200E-01 -1.629660219626E+00
P 3.153982004585E-02 -1.413699735624E-01 -1.541681808655E+00
P 2.044136251129E-01 -3.938993330674E-01 4.513572317022E-01
P 1.971779412316E-01 -2.985488231720E-01 -1.661139482239E+00
P 3.599350956262E-01 -4.829554427017E-02 4.428234315507E-01
P 3.665043910887E-01 3.563495488464E-01 -1.699416223295E+00
P 3.658280003728E-01 4.444620835775E-01 4.287589812698E-01
P 3.643388571144E-01 -1.422838043175E-01 -1.681897462501E+00
P 2.962717413853E-01 -1.803836658806E-01 6.373146063409E+00
P 1.306392962930E-01 -2.319587084687E-02 6.492694662619E+00
P 1.234010245091E-01 7.215295175768E-02 4.380176516731E+00
P 2.954484528912E-01 2.278257294028E-01 4.347341514252E+00
P 4.628112561292E-01 -2.293793099835E-02 6.447312191481E+00
P 4.641266590198E-01 7.355535392161E-02 4.355579582046E+00
P 2.960967824753E-01 3.183411036543E-01 6.484258091413E+00
P 1.309437498098E-01 4.773177447741E-01 6.532137301183E+00
P 1.287991119334E-01 -4.368152141850E-01 4.396874252063E+00
P 2.973612616456E-01 -2.811196730359E-01 4.261930324936E+00
P 4.624966325950E-01 4.778744009831E-01 6.506975265758E+00
P 4.664321279726E-01 -4.343769817188E-01 4.371235782769E+00
P -3.702204908301E-01 -1.794672220081E-01 6.459847066891E+00
P -3.697681910946E-01 2.317798789564E-01 4.393550417109E+00
P -3.698989123358E-01 3.219914930110E-01 6.516902758767E+00
P -3.696080907171E-01 -2.723634889624E-01 4.349317189049E+00
P -3.025781568367E-01 -4.936872491698E-02 4.825200920796E-01
P -3.022904757847E-01 3.562497291762E-01 -1.685303456163E+00
P -1.386091685159E-01 1.126289426043E-01 4.604111152199E-01
P -1.346825620410E-01 2.003765426797E-01 -1.675343286209E+00
P -3.024212962969E-01 4.464728426035E-01 4.380235139381E-01
P -3.019655563509E-01 -1.423076763630E-01 -1.628562218595E+00
P -1.363111637673E-01 -3.953064565340E-01 4.759155478212E-01
P -1.349925044939E-01 -2.988226284421E-01 -1.615845778477E+00
P -3.651595739662E-02 -1.794700739179E-01 6.513385109836E+00
P -2.028980180021E-01 -2.188628716391E-02 6.461166561387E+00
P -2.030538446789E-01 7.390627590689E-02 4.349968679631E+00
P -3.801610012403E-02 2.337865154736E-01 4.402849813091E+00
P -3.869069733611E-02 3.218917156601E-01 6.531038885017E+00
P -2.033981988303E-01 4.811353941900E-01 6.493151052425E+00

P -2.028696269295E-01 -4.286671280160E-01 4.356400725178E+00
P -3.211583840374E-02 -2.734515561843E-01 4.388664970757E+00
Pd 3.445551082411E-02 -9.747260001592E-02 2.874147707137E+00
Pd 2.933992320168E-01 -2.242790674432E-01 1.957469506374E+00

Energy : -16638.054983797 AU

***Optimized structure of the $Pd_2$/BP adduct with $Pd_2$ unit on top of the surface (Figure S11).***

CELL 10.04047815 9.47181658 89.998985

P 7.988274922215E-02 -1.232200532576E-01 5.980546522355E-01
P -4.193821364289E-01 3.420265479416E-02 5.205246272182E-01
P -4.105582135548E-01 1.281169335421E-01 -1.598711391900E+00
P 7.916366638338E-02 2.939485535128E-01 -1.479028328842E+00
P 2.459551994544E-01 3.422982318410E-02 5.194722137516E-01
P 2.371792972516E-01 1.281288878820E-01 -1.599684141298E+00
P 8.024709723617E-02 3.805706783430E-01 6.508667189463E-01
P -4.239135721298E-01 -4.666062815608E-01 5.922055065142E-01
P -4.209691815325E-01 -3.772048120534E-01 -1.537374766985E+00
P 7.990230254767E-02 -2.214677025673E-01 -1.515054020139E+00
P 2.504990201963E-01 -4.666245997169E-01 5.910082981025E-01
P 2.475611996118E-01 -3.772072106093E-01 -1.538402739221E+00
P 4.132721340525E-01 -1.218990509916E-01 5.191697653731E-01
P 4.133098250562E-01 2.806724313367E-01 -1.614911535088E+00
P 4.132954167516E-01 3.708770393249E-01 5.319497203766E-01
P 4.132993229026E-01 -2.184475029753E-01 -1.573058550593E+00
P 2.468188335562E-01 -9.872158611844E-02 6.380741254869E+00
P 8.022164823514E-02 5.880213230164E-02 6.377546502560E+00
P 8.021175144901E-02 1.512830208114E-01 4.259925718148E+00
P 2.469430201851E-01 3.086644703700E-01 4.265919842259E+00
P 4.135320146136E-01 5.866201840848E-02 6.379396712552E+00
P 4.134887432203E-01 1.511364888480E-01 4.262070871953E+00
P 2.468565464776E-01 4.011053197305E-01 6.384185122909E+00
P 8.018838402740E-02 -4.414056972381E-01 6.383665275856E+00
P 8.016608368005E-02 -3.489299639576E-01 4.265887493249E+00
P 2.469275364654E-01 -1.916437632997E-01 4.265179084977E+00
P 4.135332665381E-01 -4.414058867209E-01 6.384648692760E+00
P 4.134992041097E-01 -3.491348751817E-01 4.266006468083E+00
P -4.197712878177E-01 -9.873810269103E-02 6.379738012561E+00
P -4.199430207832E-01 3.086376241713E-01 4.265761130310E+00
P -4.198156293807E-01 4.010703129138E-01 6.384041918665E+00
P -4.199420340289E-01 -1.916260373208E-01 4.264005895715E+00
P -2.532734744406E-01 -1.232141537476E-01 5.988865656603E-01
P -2.526135184458E-01 2.940016597262E-01 -1.478222201273E+00
P -8.669534737315E-02 3.416058552941E-02 5.623885450140E-01
P -8.675768262564E-02 1.326785436024E-01 -1.570792382505E+00
P -2.536034651136E-01 3.806334682949E-01 6.518525296789E-01
P -2.533407840672E-01 -2.214857210001E-01 -1.514004339979E+00
P -8.665782490433E-02 -4.612884882412E-01 6.223121133225E-01
P -8.671513091944E-02 -3.785660003335E-01 -1.521426706645E+00
P -8.649092094681E-02 -9.859759197455E-02 6.381709874728E+00
P -2.531988820590E-01 5.880029970826E-02 6.377436361585E+00
P -2.531741019536E-01 1.512879213470E-01 4.259603140159E+00
P -8.647848517314E-02 3.086971124435E-01 4.267365307555E+00
P -8.650582857757E-02 4.010977092735E-01 6.385227026642E+00
P -2.531926403627E-01 -4.414066389467E-01 6.382665289612E+00

P -2.531498483635E-01 -3.489057621400E-01 4.264111925103E+00
P -8.647936970775E-02 -1.914878441706E-01 4.266355850470E+00
PD 6.478366537013E-02 -1.735487919493E-02 -2.780358768030E+00
PD -2.388155479035E-01 -1.727548339053E-02 -2.778656156977E+00

Energy : -16637.99526720 AU

An important feature of phosphorene among other 2D materials is its band gap dependence from the number of stacked layers, ranging from the upper limit of *ca.* 2.2 eV in the monolayer to *ca.*0.35 eV in the bulk material. Such band gap can be tuned also via covalent functionalization with Lewis acidic species. In the present case, B3LYP-DFT[S12,S13] calculations predicted a band gap of *ca.* 2.16 eV for double layer naked phosphorene, which reduces to 1.86 eV upon incorporation of a $Pd_2$ unit. This can be reasonably accounted for the presence of filled Pd d-levels as well as the Pd–Pd interaction at the top of the valence band. Since the conduction band is expected to remain almost unaltered, the net result is a decreased band gap.

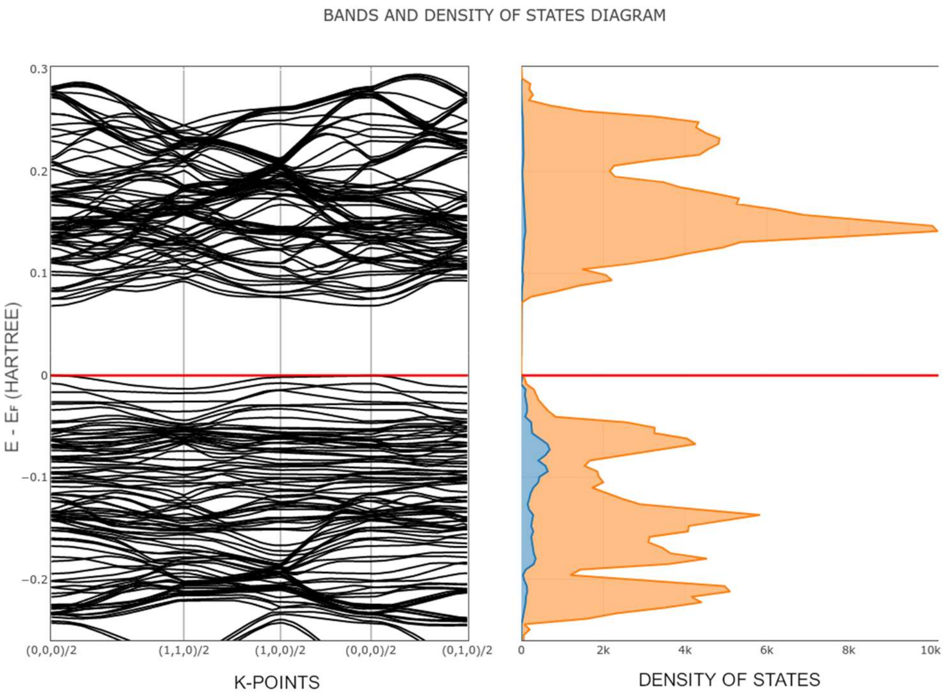


**Figure S23.** Band structure and DOS of the $Pd_2$/BP interlayer adduct, with the contribution from Pd filled d orbitals and Pd–Pd bonding highlighted in blue.

# SI References


(S1) Naumkin, A. V.; Kraut-Vaas, A.; Gaarenstroom, S. W.; Powell, C. J.NIST X-ray PhotoelectronSpectroscopy Database, NIST Standard Reference Database N. 20, National Institute of Standards andTechnology, Gaithersburg MD,2000,20899.DOI:10.18434/T4T88K

(S2) Vanni, M.; Serrano-Ruiz, M.; Telesio, F.; Heun, S.; Banchelli, M.; Matteini, P.; Mio, A. M.; Nicotra, G.; Spinella, C.; Caporali, S.; Giaccherini, A.; Acapito, F. D.; Caporali, M.; Peruzzini, M. Black Phosphorus/Palladium Nanohybrid: Unraveling the Nature of P − Pd Interaction and Application in Selective Hydrogenation. *Chem. Mater.* **2019**, *31*, 5075−5080.

(S3) Köpf, M.; Eckstein, N.; Pfister, D.; Grotz, C.; Krüger, I.; Greiwe, M.; Hansen, T.; Kohlmann, H.; Nilges, T. Access and in Situ Growth of Phosphorene-Precursor Black Phosphorus. *J. Cryst. Growth* **2014**, *405*, 6–10.

(S4) Serrano-Ruiz, M.; Caporali, M.; Ienco, A.; Piazza, V.; Heun, S.; Peruzzini, M. The Role of Water in the Preparation and Stabilization of High-Quality Phosphorene Flakes. *Adv. Mater. Interfaces* **2016**, *3*, 1500441.

(S5) Tatsuno, Y.; Yoshida, T.; Otsuka, S.; Al-Salem, N.; Shaw, B. L. ($\eta^3$-Allyl)Palladium(II) Complexes. *Inorg. Synth.* **1990**, *28*, 342– 345.

(S6) Usui, K.; Oishi, S.; Hiro, T.; Arai, T. Process for Producing Allyl Chloride. US Patent WO 2001/060772, 2002.(S7) Briois, V.; Fonda, E.; Belin, S.; Barthe, L.; La Fontaine, C.; Langlois, F.; Ribbens, M.; Villain, F. SAMBA: The 4-40 KeV X-Ray Absorption Spectroscopy Beamline at SOLEIL. *UVX 2010 - 10e Colloq. sur les Sources Coherentes Incoherentes UV, VUV X Appl. Dev. Recent.* **2011**, *2010*, 41–47.

(S8) Ravel, B.; Newville, M. ATHENA, ARTEMIS, HEPHAESTUS: Data Analysis for X-Ray Absorption Spectroscopy Using IFEFFIT. *J. Synchrotron Radiat.* **2005**, *12*, 537–541.

(S9) Ankudinov, A.; Ravel, B.; Rehr, J. J.; Conradson, S. D. Real-Space Multiple-Scattering Calculation and Interpretation of X-Ray-Absorption Near-Edge Structure. *Phys. Rev. B - Condens. Matter Mater. Phys.* **1998**, *58*, 7565–7576.

(S10) Tada, M.; Sasaki, T.; Iwasawa, Y. Design of a Novel Molecular-Imprinted Rh-Amine Complex on $SiO_2$ and its Shape-Selective Catalysis for α-Methylstyrene Hydrogenation. *J. Phys. Chem. B* **2004**, *108*, 2918–2930.

(S11) Trovitch, R. J.; Guo, N.; Janicke, M. T.; Li, H.; Marshall, C. L.; Miller, J. T.; Sattelberger, A. P.; John, K. D.; Thomas Baker, R. Spectroscopic Characterization of Alumina-Supported Bis(Allyl)Iridium Complexes: Site-Isolation, Reactivity, and Decomposition Studies. *Inorg. Chem.* **2010**, *49*, 2247–2258.

(S12) Lee, C.; Wang, W.; Parr, R. G. Development of the Colle-Salvetti correlation-energy formula into a functional of the electron density. *Phys. Rev. B: Condens. Matter* **1988**, *37*, 785-789.

(S13) Becke, A. D. Density-functional thermochemistry. III. The role of exact exchange. *J. Chem. Phys.* **1993**, *98*, 5648-5652.

(S14) Dovesi, R.; Erba, A.; Orlando, R.; Zicovich-Wilson, C. M.; Civalleri, B.; Maschio, L.; Rérat, M.; Casassa, S.; Baima, J.; Salustro, S.; Kirtman, B. Quantum-Mechanical Condensed Matter Simulations with CRYSTAL. *Wiley Interdiscip. Rev. Comput. Mol. Sci.* **2018**, e1360.

(S15) Hay, P. J.; Wadt, W. R. Potentials for K to Au Including the Outermost Core Orbitals. *J. Chem. Phys* **1985**, *82*, 299–310.
